# Sub-stoichiometric 5-methoxyuridine modification enables tunable immune evasion and protein expression from synthetic mRNAs

**DOI:** 10.1016/j.omtn.2026.103058

**Published:** 2026-08-12

**Authors:** Jonathan R. Miles, Sarah Masterson, Eleanor Bellows, Fabio Fisher, Ritu Rani, Sara Marelli, Colin Hardman, Luigi Grassi, Luis Santos, Victoria James, Nigel P. Mongan, Christopher J. Hayes, Matthew Loose, Janet M. Daly, James Button, George Thom, Rupert G. Fray, Nathan Archer

**Affiliations:** 1 School of Veterinary Medicine and Science, University of Nottingham, Sutton Bonington Campus, LE12 5RD Leicestershire, UK; 2 Biodiscovery Institute, University of Nottingham, NG7 2RD Nottingham, UK; 3 Biopharmaceuticals R&D, Discovery Sciences, Nucleic Acid Therapeutics, RNA Therapy Team, AstraZeneca, CB2 0AA Cambridge, UK; 4 Biopharmaceuticals R&D, Data Science & Advanced Analytics, Data Science and AI, AstraZeneca, CB2 0AA Cambridge, UK; 5 Biopharmaceuticals R&D, Biopharmaceutical Development, AstraZeneca, CB2 0AA Cambridge, UK; 6 Biopharmaceuticals R&D, Biopharmaceutical Development, AstraZeneca, Gaithersburg, MD 20878, USA; 7 Weill Cornell Medicine, New York, NY 10065, USA; 8 School of Chemistry, University of Nottingham, NG7 2RD Nottingham, UK; 9 School of Life Sciences, University of Nottingham, NG7 2RD Nottingham, UK; 10 School of Biosciences, University of Nottingham, Sutton Bonington Campus, LE12 5RD Leicestershire, UK

**Keywords:** MT: oligonucleotides: therapies and applications, epitranscriptome, mRNA therapeutics, 5-methoxy-uracil

## Abstract

mRNA therapeutics depend on nucleotide modification to evade innate immunity and sustain translation. Current clinical designs replace all uridine with N1-methylpseudouridine (m1Ψ). However, full substitution is costly and can perturb translational fidelity and RNA structure. Here, we show that sub-stoichiometric incorporation of 5-methoxyuridine (5moU) is sufficient to confer immune evasion while enhancing protein output. Substitution of 25% of uridines with 5moU supported translation comparable to fully m1Ψ-modified mRNA in some cell types, while 50% 5moU maximized expression across all systems tested, often outperforming 100% m1Ψ-modified mRNA. This effect was conserved in human primary cells and *in vivo*, where lipid nanoparticle-delivered mRNA encoding human IgG produced approximately 2-fold higher serum levels with 50% 5moU than with 100% m1Ψ. Transcriptomic profiling revealed reduced interferon-stimulated gene induction following delivery of 5moU-modified mRNA compared with unmodified and m1Ψ modified mRNA. Unlike m1Ψ, 5moU modification consistently suppressed innate responses to double-stranded RNA. Together, these data demonstrate that partial nucleotide substitution can outperform current mRNA modification strategies and expand the design space for therapeutic mRNAs.

## Graphical abstract

**Figure:**
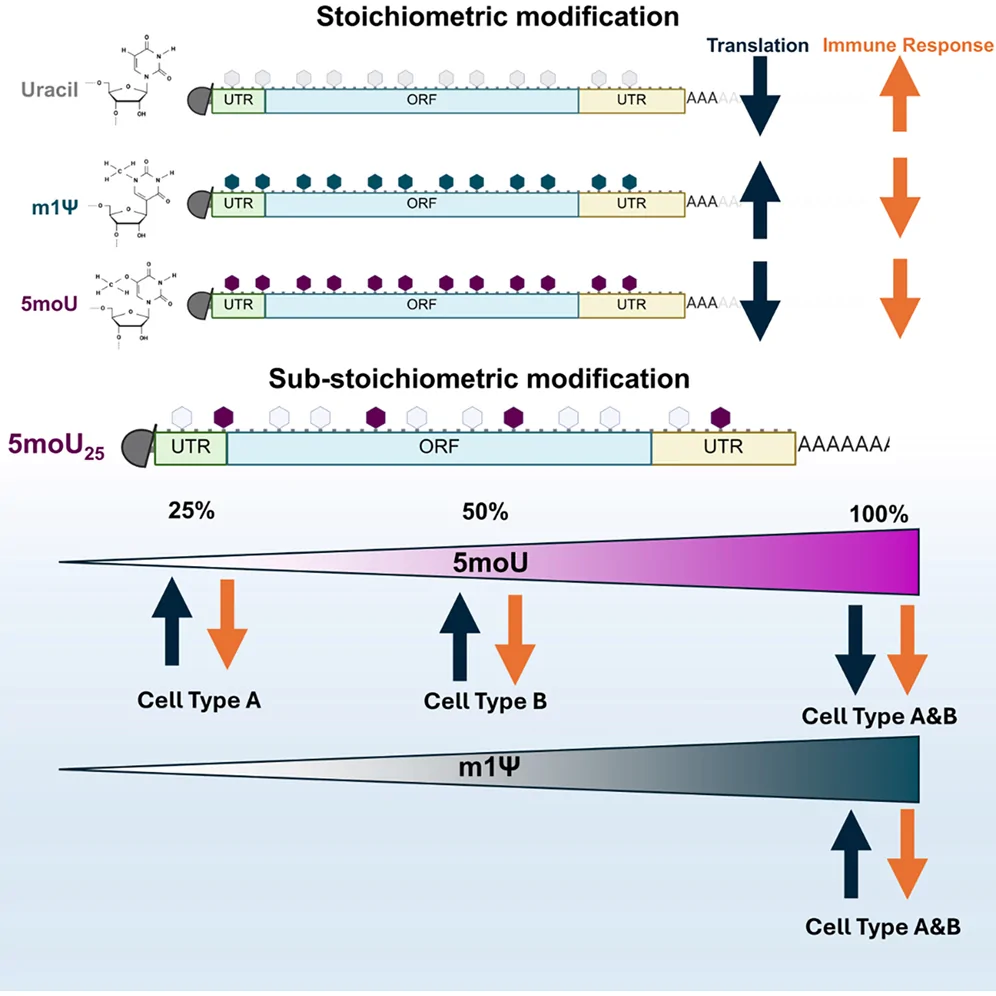

## Teaser

Current mRNA therapeutics typically rely on complete substitution of uridine with a modified nucleotide, most commonly N1-methylpseudouridine. Archer and colleagues show that partial 5-methoxyuridine incorporation suppresses innate immune sensing while increasing protein output, in a cell-type-specific manner. These findings establish modification stoichiometry as a tunable design principle for mRNA medicines.

## Introduction

The ability to deliver RNA encoding antigens or therapeutic proteins to cells has transformed strategies for treating cancer, infectious diseases, and genetic disorders.^1^ Messenger RNA (mRNA) therapies offer a uniquely adaptable platform, enabling rapid redesign, scalable manufacturing, and efficient intracellular delivery. Clinical applications currently under investigation include personalized neoantigen-based cancer vaccines,^2^ delivery of functional proteins for conditions such as cystic fibrosis (CFTR),^3^ and treatment of rare diseases such as argininosuccinic aciduria.^4^ Delivery of mRNA encoding antibodies also offers the potential to confer rapid, albeit transient, resistance to infection.^5^ As these platforms mature, improving the potency, safety, and controllability of nucleic acid-based drugs has become a central design challenge.^6^

A central hurdle in mRNA therapeutics is evading recognition by the innate immune system. Immune activation limits efficacy by promoting RNA sequestration, translational repression, and degradation.^7^ Early work by Kariko and colleagues^8^ demonstrated that replacing canonical nucleotides with pseudouridine (Ψ), *N*6-methyladenosine (m^6^A), or 5-methylcytosine (m^5^C) reduces innate immune response to the foreign RNA. In their work, 100% substitution of canonical bases abrogated the immune response in monocyte-derived dendritic cells.^8^^,^^9^ Incomplete and mixed modifications demonstrated early promise in mouse models congenital surfactant protein B (SP-B) deficiency,^10^ but subsequent studies identified N1-methylpseudouridine (m1Ψ) as a particularly effective modification,^11^ capable of evading type I interferon responses and preventing activation of pattern recognition receptors such as *RIG-I* and Toll-like receptors (TLRs).^8^^,^^12^^,^^13^ m1Ψ has since become the dominant internal modification used in mRNA vaccines and therapies, including those developed for SARS-CoV-2. Cap modifications^14^^,^^15^^,^^16^^,^^17^ and lipid nanoparticle encapsulation^18^ are also key areas of development.

Although m1Ψ-substitution is effective at increasing ribosome loading onto RNA, thereby increasing its translational potential, it can also induce frameshifting and ribosome pausing.^19^ m1Ψ also affects secondary structure,^20^ making it unsuitable for some RNA therapeutic platforms. Given the diversity of naturally occurring RNA modifications,^21^ the chemical design space for mRNA therapeutics remains largely unexplored.

Optimal mRNA design varies by application. Gene therapies prioritize sustained, immune-silent expression, whereas vaccines favor transient expression and may benefit from the innate immune response to the mRNA itself.^22^ In either case, engineering cell-type specificity is of significant interest, for example recent work targeting antigen-presenting cells in the spleen for vaccination^23^ and *in vivo* CAR-T therapy^24^

Here, we investigate 5-methoxyuridine (5moU). Prior work has found 5moU modification to be very effective at reducing innate immune activation following delivery to cells.^25^^,^^26^ However, complete substitution with 5moU has mixed results.^19^^,^^27^^,^^28^^,^^29^ In Hep 3B and THP-1 cells, 5moU ranked third of seven modifications for productivity,^28^ while in HeLa cells, 5moU appears consistently poor.^19^^,^^29^ Early studies by Karikó, Weissman, and others tested substoichiometric nucleotide modifications and found it insufficient, helping cement a paradigm of full nucleotide replacement. However, the epitranscriptome revolution has unlocked many natural and unnatural nucleotide modifications, and so we sought to test the hypothesis that partial (sub-stoichiometric) nucleotide modification may improve the properties of 5moU-modified synthetic mRNA.

Here, we assess the translational capacity of RNAs containing 25%, 50%, or 100% substitution with 5moU (5moU_25_, 5moU_50,_ and 5moU_100_) and compare this to equivalent substitution levels of m1Ψ across *in vitro* and *in vivo* systems*.* By integrating analyses of protein output, innate immune responses, and transcriptomic responses across human cell lines and primary cells and by assessing protein expression in mice following delivery of an mRNA encoding human immunoglobulin (hIgG), we define the cell-type-specific potential of sub-stoichiometric mRNA modification. Together, this work establishes sub-stoichiometric nucleotide incorporation as a generalizable framework for tuning mRNA behavior, adding a new dimension to therapeutic RNA design.

## Results

### Partial uridine substitution with 5moU improves translation and immunogenic properties over total substitution with m1Ψ

**Figure 1:**
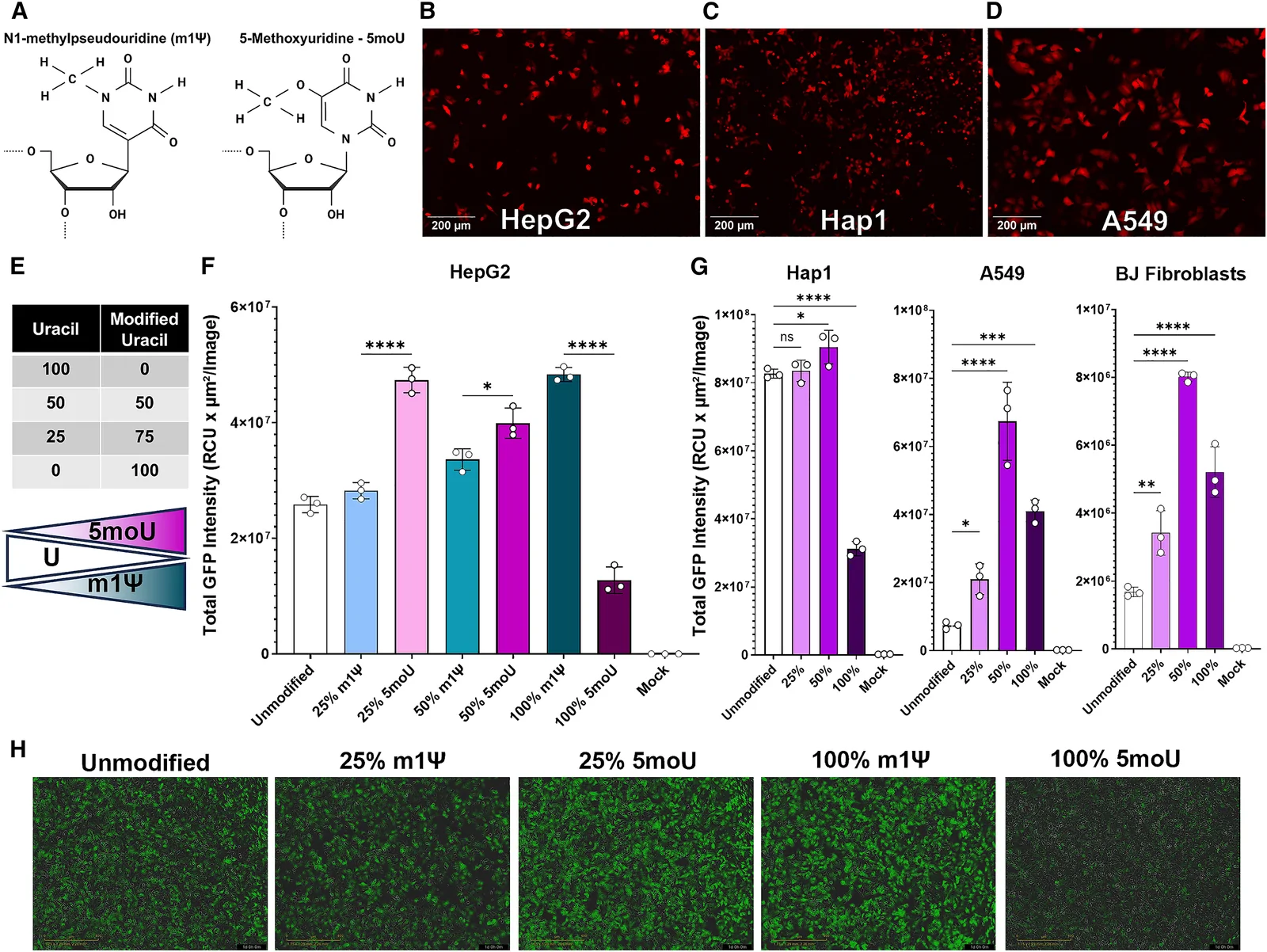
5moU confers immune evasion and efficient translation to mRNA under partial substitution (A) 5moU is a natural modification found on tRNA, where the 5′ carbon of the uridine base is methoxylated. (B–D) Fluorescent microscopy after transfection of completely 5moU-modified mRNA encoding mCherry fluorescent reporter in HepG2 (B), Hap1 (C), and A549 (D) cells. (E) Sub-stoichiometric modification percentages tested for 5moU and m1Ψ. (F) Total GFP intensity measured 24 h post-transfection for each of the eGFP-mRNAs tested in HepG2 cells for 5moU and m1Ψ. (G) Total GFP intensity measured 24 h after transfection of 5moU-modified mRNA into Hap1, A549, and BJ fibroblast cell lines. (H) Fluorescent and light (composite) microscopy images collected by the incucyte at 24 h time point comparing unmodified, m1Ψ_25,100_, and 5moU_25,100_ mRNA in HepG2 cells. Significance: ∗*p* < 0.05, ∗∗*p* < 0.01, ∗∗∗*p* < 0.001, ∗∗∗∗*p* < 0.0001. *N* = 3 for (F)‑(G). a.u., arbitrary units

Prior work found that 5moU-modified mRNA is poorly translated in HeLa cells.^19^ To test if this were true for other cell types, we assayed translation of 5moU_100_ across various cell types: HepG2, derived from a human hepatocellular carcinoma; Hap1, a near-haploid human cell line derived from KBM-7, a chronic myeloid leukemia line; and A549, from a human lung carcinoma of epithelial origin. We transfected a commercially available mRNA encoding mCherry, 5moU_100_, and Cap1 modified mRNA into these cells, finding translation to be consistently detectable by fluorescent microscopy (Figures 1B–1D).

Early work established the current paradigm in which mRNA therapeutics are designed with total substitution.^8^^,^^9^^,^^13^ However, dose- and cell-type-dependent relationships have not been well studied with other modified nucleotides. We, therefore, questioned whether the impact of 5moU on translation was proportional to the quantity of 5moU incorporated in a transfected RNA.

We tested the proportional incorporation and uniform distribution by combining two-dimensional thin layer chromatography and Oxford-nanopore based direct-RNA sequencing (DRS) (Figures S1–S4). An adapted buffer system separated 5moU from unmodified uracil, and densitometry confirmed that 5moU signal increased proportionally with the percentage of 5moU included during *in vitro* transcription (IVT) (Figures S1A–S1C). There are a variety of tools for the quality control of therapeutic mRNAs by DRS. However, existing DRS workflows for vaccine mRNA aim to identify off-target transcription products and estimate poly(A) tail length,^30^ but they are not designed to analyze RNA with less than 100% nucleotide substitution. To fill the gap created by “sub-stoichiometric” modification, we developed a workflow using DRS (Figure S1D). We first extracted and queried the error rates of each position in the basecalled data using Epinano^31^ (Figure S1E). Mismatch rates clearly differentiated between unmodified and 5moU mRNAs (Figures S1E–S1H). Capitalizing on this, we were able to plot the delta mismatch ratio across the target RNAs to examine the *in vitro* transcripts for trends in incorporation. We performed this analysis on long *in vitro* transcribed mRNAs carrying distinct 5′ UTRs and coding sequences, testing for effects of transcript length-, sequence-, or UTR-dependent biases in 25% 5moU incorporation (Figures S1I–S1K); 5moU incorporation was broadly uniform across the transcript length.

We then transcribed mRNAs encoding enhanced green fluorescent protein (eGFP), substituting 25%, 50%, or 100% of uridine in the reaction mix with either 5moU or m1Ψ to generate sub-stoichiometric-modified mRNA (Figure 1E). We confirmed the same broad distribution of 5moU in the 5moU_25_ eGFP mRNA with our nanopore pipeline (Figure S4). We first transfected HepG2 cells and monitored expression over time. At 24 h and as expected, m1Ψ-modified mRNA translation peaked at 100% substitution. In contrast, 5moU-modified mRNA translation peaked at 25% substitution, at which it matched the output of 100% m1Ψ (Figure 1F).

Given that other studies exploring 5moU_100_ found poor or no expression in some cell types, we tested 5moU_25-100_ across our panel of cells, adding BJ fibroblasts, derived from neonatal foreskin, to represent non-transformed, stromal cells. We found variations in the impact of 5moU-modification by cell type; Hap1 cells had minor increases in translation at 50% substitution compared with unmodified, whereas translation in A549 and BJ fibroblast cells was greatly increased. Notably, none of the cell types tested exhibited maximal expression at 100% substitution, while in both HepG2 and Hap1 cells, total substitution with 5moU resulted in poorer expression than completely unmodified mRNA. The differences in HepG2 cells were readily observed by fluorescent microscopy (Figure 1H). We then compared 100% m1Ψ modification with 50% 5moU in A549 cells directly, and at 24 h, m1Ψ_100_ mRNA exhibited higher expression than 5moU_50_ mRNA, but this was not statistically significant (Figure S5).

### Sub-stoichiometric modification of mRNA with 5moU confers immune evasion

**Figure 2:**
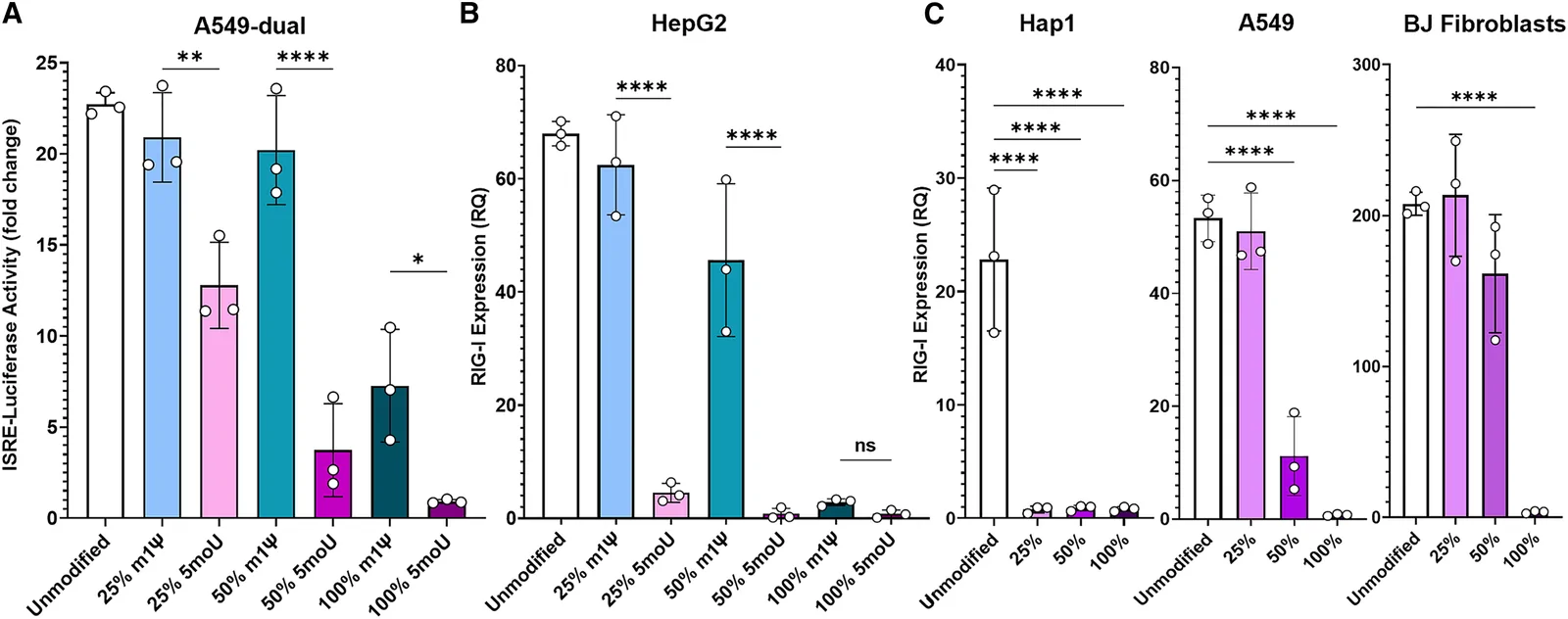
5moU modification elicits earlier immune silencing at a lower stoichiometry than m1Ψ (A) Immunogenicity of the modified mRNAs was measured using A549-Dual cells following transfection with modified mRNAs, 24 h before detection of interferon-stimulated response element (ISRE) activation by luciferase assay of the cell media. (B–C) qPCR-measured RQ expression of RIG-I in (B) HepG2 following transfection of 5moU and m1Ψ sub-stoichiometric mRNA, and (C) Hap1, A549, and BJ fibroblast cell lines following transfection of 5moU sub-stoichiometric mRNA. Significance: ∗*p* < 0.05, ∗∗*p* < 0.01, ∗∗∗*p* < 0.001, ∗∗∗∗*p* < 0.0001. Calculated by ordinary one-way ANOVA followed by Tukey’s multiple comparison test. Error bars show standard deviation. (*N* = 3).

Limiting the immune response to therapeutic mRNA is largely considered to be the key engineering challenge. The innate immune response leads to RNA sequestration, degradation, and dampening of translation, thereby reducing production of the therapeutic protein. To characterize the immunogenicity of 5moU_25-100_ mRNA, we first used A549-Dual cells, which express Lucia luciferase under an interferon response factor (IRF)-regulated promoter. Secreted Lucia luciferase in the growth media then serves as a readout of innate immune activation. After transfection with A549-Dual cells with our mRNA reporters, immunogenicity was quantified (Figure 2A). As expected, the immunogenic response to m1Ψ-modified RNA was only significantly reduced at 100% (stoichiometric) substitution. However, 5moU significantly attenuated the immune response at sub-stoichiometric levels, and at 50%, it was more effective than total replacement with m1Ψ_100_. We further profiled the immune responses in our panel of cell lines with RT-qPCR for *RIG-I* expression as well as *IFIT1* and *MDA5* (Table S4). In HepG2 cells, m1Ψ-mediated immune evasion again required 100% substitution (Figure 2B), while sub-stoichiometric modification with 5moU was effective. Immune evasion was achieved at 25% substitution in HepG2 and Hap1 cells, 50% in A549 cells, but only at 100% substitution in BJ fibroblasts (Figure 2C).

### 5moU outperforms m1Ψ *in vivo* and with *ex vivo* human cells

**Figure 3:**
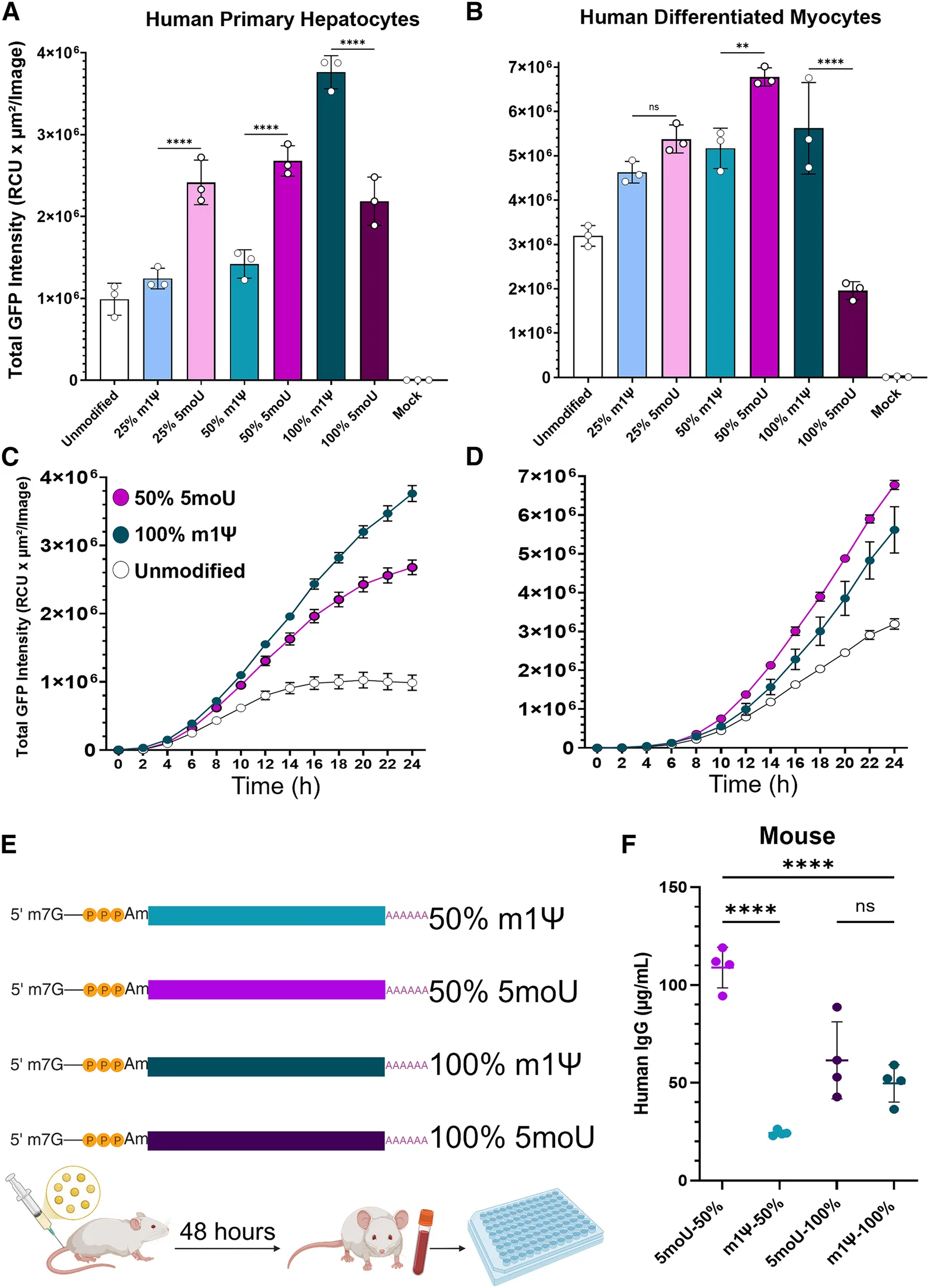
5moU_50_ mRNA offers improved translation in *ex vivo* human cells and for IgG production *in vivo* (A–D) Total GFP intensity following transfection with substoichiometric mRNA encoding eGFP. Primary human hepatocytes (A) and differentiated myocytes (B) 24 h after transfection (*N* = 3), and across the time course for 5moU_50_ and m1Ψ_100_ modified mRNA (C and D). (E) Schematic: m1Ψ_50,_ m1Ψ_100,_ 5moU_50_, and 5moU_100_ mRNA transcripts encoding heavy and light chains of human IgG were LNP-encapsulated and delivered to mouse, with sera evaluated after 48 h for antibody concentration. (F) Blood IgG concentrations (μg/mL) measured by ELISA (*N* = 4). Significance: ∗*p* < 0.05, ∗∗*p* < 0.01, ∗∗∗*p* < 0.001, ∗∗∗∗*p* < 0.0001. Calculated by ordinary one-way ANOVA followed by Tukey’s multiple comparison test. (A, B, and F) Error bars show SD. (C and D) Error bars show SEM.

To test whether the performance and cell-type-specific effects observed *in vitro* extend to more physiologically relevant systems, we evaluated 5moU-modified mRNA in *ex vivo* human hepatocytes and skeletal muscle cells. Human hepatocytes and differentiated skeletal myocytes were transfected with our sub-stoichiometric reporter mRNA encoding eGFP and fluorescence recorded over time (Figures 3A–3D). Translation remained tissue-specific, with hepatocytes maximally translating m1Ψ_100_ over the others tested (Figure 3A). By contrast, myocytes translated 5moU_50_ maximally, with m1Ψ having more limited expression (Figure 3B). In both cell types, 100% 5moU inhibited translation. Translation of 5moU_50_ plateaued in the human primary hepatocytes (Figure 3C), but no such plateau is visible for m1Ψ_100_-eGFP or 5moU_50_-eGFP mRNA in differentiated myocytes (Figure 3D). These differences in expression over time reflect cell type differences observed *in vitro* across the time course (Figure S6). In myocytes, m1Ψ_50_-eGFP translation plateaued around the 14 h time point (Figure S6C).

To assess whether sub-stoichiometric modification improves protein output *in vivo*, we evaluated delivery and expression of hIgG in mice using lipid nanoparticle (LNP)-encapsulated mRNAs encoding IgG heavy (HC) and light (LC) chains. Transcripts were modified with either 50% or 100% 5moU, or with equivalent substitution levels of m1Ψ. Following intravenous administration, serum hIgG concentrations were quantified by ELISA 48 h post-injection. Mice receiving fully substituted mRNAs produced comparable levels of circulating antibody, with serum hIgG reaching 50–60 μg/mL for both 100% m1Ψ and 100% 5moU. Reducing m1Ψ substitution to 50% more than halved antibody output to 24 μg/mL. In contrast, 50% 5moU-modified mRNA was 4.5-fold more productive, yielding 109 μg/mL hIgG and exceeding expression achieved with 100% m1Ψ-modified mRNA by 1.75-fold (Figure 3F).

### 5moU incorporation enables mRNA to evade the TLR3 cascade and innate immunity

To define whether 5moU-modified mRNA engages cellular response pathways distinct from those triggered by m1Ψ-modified mRNA, we profiled transcriptomic changes following transfection and compared these responses with those elicited by unmodified mRNA. HepG2 cells were transfected with unmodified, m1Ψ_100_, or 5moU_25_ eGFP mRNA and subjected to RNA-sequencing 24 h after transfection.

**Figure 4:**
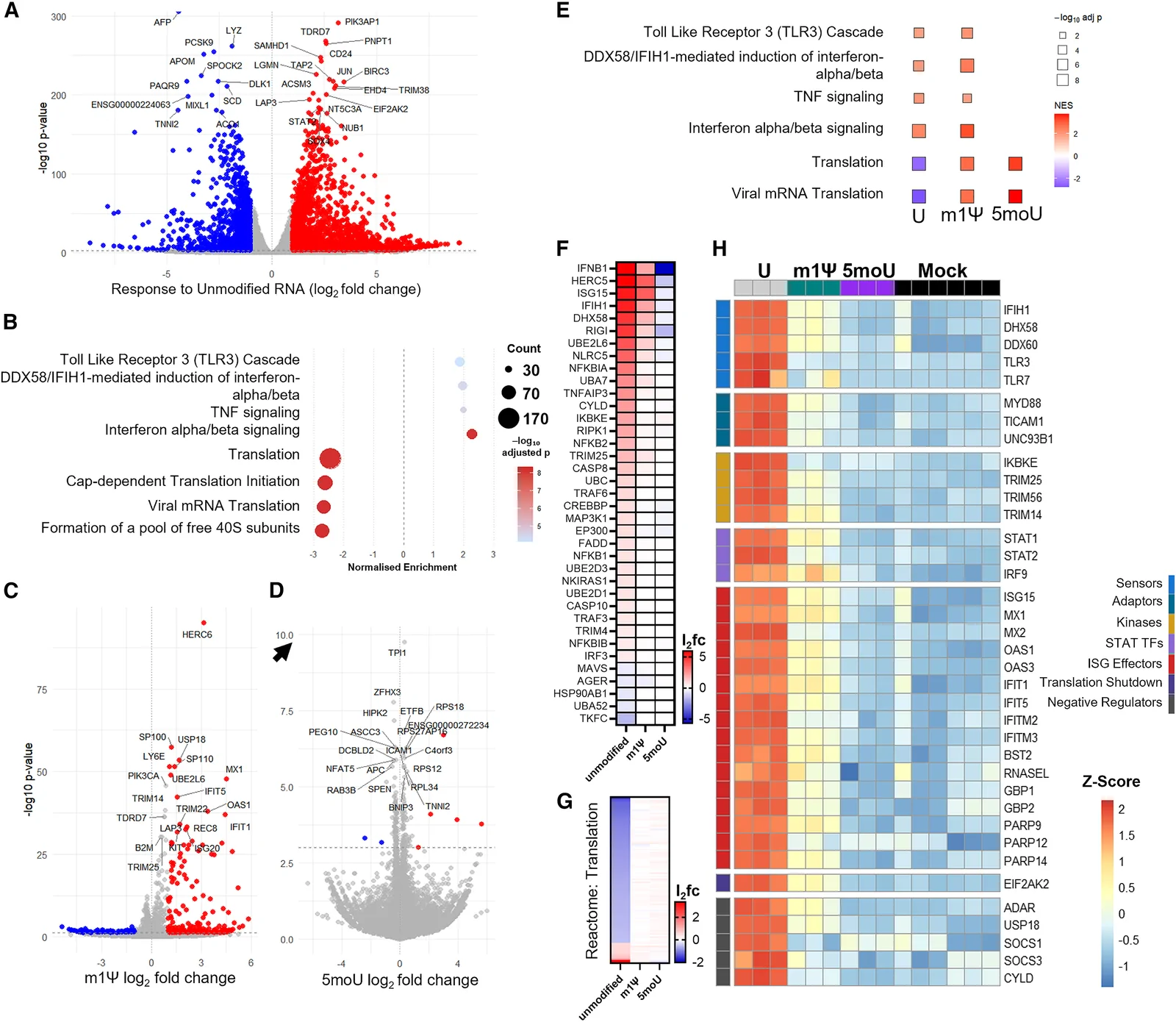
Substoichiometric 5moU modification enables broad evasion of the cellular innate immune response (A) Volcano plot showing differential gene expression following transfection of HepG2 cells with unmodified mRNA, with upregulated genes in red and downregulated in blue. (B) Reactome gene set enrichment analysis (GSEA) performed on all genes detected, sorted by Wald statistic. (C and D) Volcano plots showing differentially expressed genes in cells responding to m1Ψ_100_ (C) or 5moU_25_ mRNA (D). (E) Bubble plot summarizing GSEA across all challenges. (F and G) Heatmap showing the log_2_-fold change for genes associated with Reactome terms *DDX/IFIH1* (MDA5) mediated induction of interferon alpha/beta (F) or translation (G); genes with a log_2_ fold change between -0.5 and +0.5 in response to unmodified mRNA were removed before plotting. (H) Genes stimulated following unmodified RNA transfection and positive in the m1Ψ_100_ response, but are not stimulated following 5moU_25_. Expression in each sequencing sample was *Z* score normalized and plotted as a heatmap.

Delivery of unmodified mRNA induced extensive transcriptional remodeling, with over 3,500 differentially expressed genes (DEGs) identified (absolute log_2_ fold change greater than 1 and an adjusted *p* value <0.01) (Figure 4A), of which 2,316 genes were upregulated. Gene Ontology (GO) enrichment analysis on upregulated genes identified numerous enriched biological processes (Table S5), including expected terms for interferon and interleukin production, as well as regulation of innate immune response and defense against viruses. Performing gene set enrichment analysis (GSEA) against Reactome terms also identified characteristic activated pathways included TLR3 cascade response and *RIG-I*/*MDA5* mediated responses, while inhibited pathways included those involved in translation (Figure 4B).

Having identified the genes regulated in response to unmodified mRNA, we performed differential gene expression analysis in cells responding to m1Ψ_100_ or 5moU_25_ (Figure 4C). Both m1Ψ_100_ and 5moU_25_ mRNA elicited much smaller responses compared to unmodified mRNA. Only 63 genes had an absolute log_2_ fold change greater than 1 and an adjusted *p* value <0.01 in response to m1Ψ_100_, with several canonical innate RNA response genes, including *IFIT1*, *PKR* (*EIF2AK2*), and *OAS1*, remaining among the upregulated genes. The response to 5moU_25_ was further reduced, with 40 genes attaining an adjusted *p* value <0.01 and none of these reaching the fold change threshold (Figures 4D; Table S5).

We then performed GSEA across our comparisons, to examine the Reactome pathways enriched in each dataset (Figures 4E; Table S5). This suggested that 5moU_25_ is particularly effective at evading the RIG-I (DDX58/IFIH1) mediated induction of interferon alpha/beta, and the TLR3 cascade, while translation terms were no longer downregulated when transfected with 5moU or m1Ψ modified mRNA. To confirm these differences, we annotated genes in the DDX58/IFIH1-mediated induction of interferon-alpha/beta (MDA5 pathway) and translation Reactome terms with their log_2_ fold change in our data (Figures 4F and 4G). While an overall weaker response, m1Ψ_100_ still elicits some changes in the dsRNA responsive, MDA5 pathway; 5moU_25_ modification appears to completely avoid triggering this pathway.

While the response to 5moU_25_ mRNA was limited, plotting individual counts for the assayed sensors shows some sensors and interferon-stimulated gene (ISG) effectors may still be upregulated in response to 5moU_25_, though still remaining much lower than m1Ψ_100_ (Figures S7 and S8). Therefore, to finalize a set of genes differentially regulated between m1Ψ_100_- and 5moU_25_-modified mRNA, we performed *Z* score analysis across all datasets, focusing on transcripts showing the greatest discordance between conditions (Figure 4H). These discordant genes are largely reflective of the enrichment data as components of the innate immune response to double-stranded RNA or other foreign RNA types.

### 5moU modification prevents innate immune responses to dsRNA

**Figure 5:**
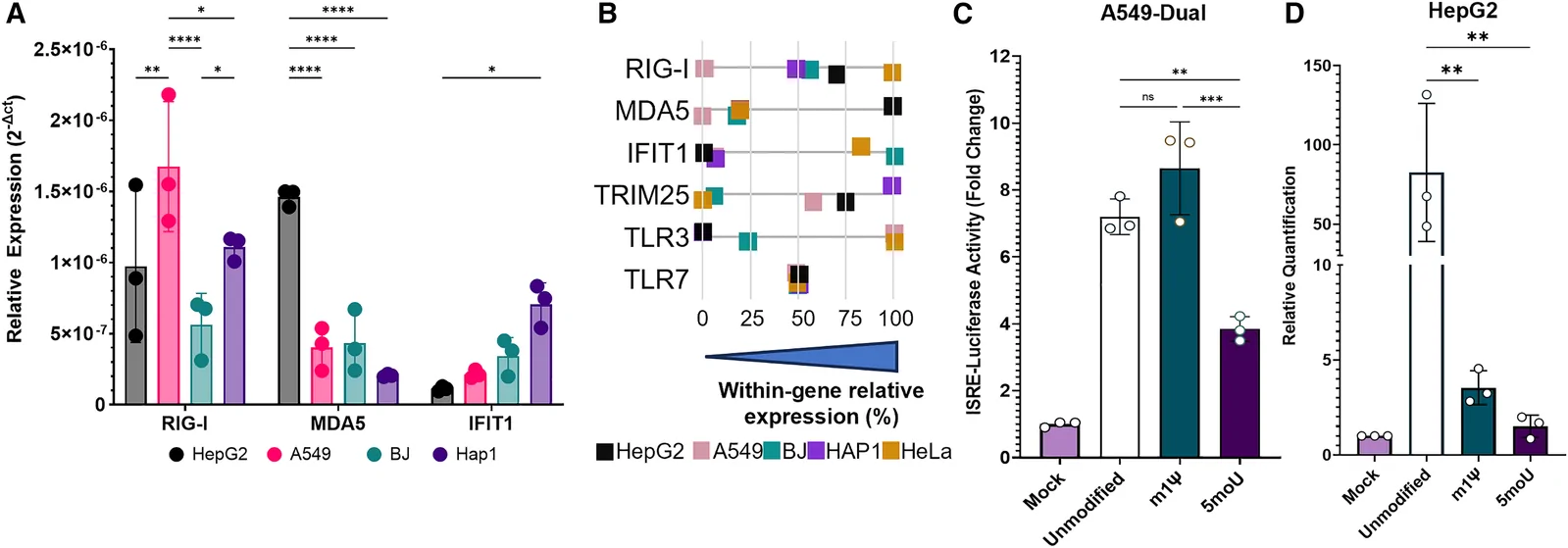
5moU modification prevents dsRNA immune response (A) Baseline expression of RIG-I, MDA5, and IFIT1 by qPCR in mock treatments from Figure 1. (B)Normalized transcript expression data from human protein atlas for select innate immune genes across HepG2, A549, BJ Fibroblast, Hap1, and HeLa cell lines. (C and D) Immune response to unmodified, m1Ψ_100_, or 5moU_100_ double-stranded RNA, as measured by luciferase activity in A549-Dual cells (C) or qPCR RIG-I RQ in HepG2 (D).

To determine whether cell-type variability in the expression of foreign RNA sensors could account for the different responses observed between cell lines, we examined the basal expression of key innate immune sensors. Revisiting the qPCR data from mock-transfected controls, we quantified expression of *RIG-I*, *MDA5*, and *IFIT1* and observed significant differences between cell types, most notably between HepG2 and A549 cells, consistent with their distinct sensitivities to 5moU-modified mRNA (Figure 5A). We extended this analysis by interrogating the normalized expression data available in the Human Protein Atlas,^32^ normalizing each gene for specific cell types enables the plotting of the relative expression of those genes identified earlier (Figure 5B). The dsRNA sensor *MDA5/IFIH1* was most consistent between these analyses.

Given these differences in innate immune sensor expression, we next asked whether 5moU modification alters cellular responses to dsRNA itself. This is a common byproduct in synthetic RNA production, but double-stranded RNA structures are also both an inherent property of RNA and a potentially powerful engineering opportunity. A dot blot for dsRNA found that while uracil modifications reduced the detectable dsRNA levels in synthesized mRNAs, there were no differences between 5moU and m1Ψ at either 25% or 100% substitution (Figure S9). To test whether the response to dsRNA is affected, we purchased unmodified, m1Ψ_100_-modified, or 5moU_100_-modified dsRNA 300 bp in length, and transfected it into A549-Dual and HepG2 cells. In A549-Dual cells, culture media were analyzed after 18 h for luciferase activity, while in HepG2 cells, total RNA was collected after 24 h for RT-qPCR analysis. In A549-Dual cells, m1Ψ_100_ modification did not prevent immune stimulation by dsRNA, whereas 5moU_100_ modification significantly reduced the cellular response (Figure 5C). In HepG2 cells, upregulation of RIG-I was prevented by both m1Ψ_100_- and 5moU_100_-modified dsRNA (Figure 5D).

## Discussion

Our findings outline a design principle for therapeutic mRNA that leverages sub-stoichiometric modification to improve protein yield, and introduce cell-type-specific behavior. We show that partial incorporation of 5moU, particularly at 25%–50%, can produce functional outcomes that in many contexts match or exceed those of m1Ψ_100_, the current benchmark for clinical mRNA production. Sub-stoichiometric 5moU_50_-modified mRNA encapsulated in LNPs enables robust *in vivo* delivery of human IgG, underscoring the translational potential of both sub-stoichiometric modification and 5moU.

Although previous studies have reported poor or inconsistent expression with 5moU-modified mRNA,^18^^,^^19^^,^^28^^,^^29^ we show that 5moU can support translation across a range of cell types and *in vivo*, when used at sub-stoichiometric levels. 5moU also exhibits sequence-specific effects. For example, in work exploring m1Ψ-induced frameshifting, a 5moU_100_ luciferase mRNA was poorly translated in HeLa cells.^19^ This was consistent with earlier findings wherein 5moU_100-_eGFP mRNA was well translated in Hep 3B and THP-1 cells, but luciferase mRNA was not.^28^ These differences suggest that both sequence context and innate immune background influence 5moU_100_ translation, and thus with our data, tuning stoichiometry is able to mitigate these effects and facilitate more precise cell-type-specific expression.

We found that 5moU exhibits distinct and cell-type-specific effects on translation and immunogenicity. Translation peaked in most cell types around 50%, and innate immune activation was attenuated broadly at lower stoichiometries (25%). Reducing the substitution ratio restored translation of modified mRNA, while retaining reduced immunogenicity. The widely used m1Ψ_100_ modification induces +1 ribosomal frameshifting,^19^ but the same study did not detect frameshifting on 5moU_100_ RNA. Further work is, therefore, required to define why complete 5moU substitution is strongly inhibitory to translation, and how this inhibition is relieved by substoichiometric incorporation.

The innate immune pathways evaded by substoichiometric 5moU modification remain unresolved. Innate immune sensing remains a major barrier for mRNA therapeutics. Here, in HepG2 cells, the delivery of unmodified RNA activated canonical RIG-I, TLR3, TNF-associated, and growth-inhibitory pathways, consistent with earlier studies.^11^^,^^33^ These responses were strongly reduced by m1Ψ_100_ and were markedly attenuated at only 25% moU incorporation. Whether the same degree of attenuation occurs (and the level of 5moU incorporation required) in immune cells with their distinct RNA-sensor expression profiles remains to be determined. In addition to pro-inflammatory pathways, cell growth inhibitory pathways (including OAS, RNAse L, and PKR-dependent pathways) are typically activated and form part of the innate blockade consistent with the reduced protein yield observed here. Recent work has shown that foreign RNA can induce growth-inhibitory pathways independently of inflammatory signaling.^33^ The same work found that while modifications could not reduce the immunogenicity of dsRNA, m6A could prevent growth inhibitory pathway activation. In contrast, our data show that a 5moU_100_ dsRNA failed to activate the innate immune response. Assessing our reporters for dsRNA contamination found no differences in dsRNA contamination relative to m1Ψ-modified RNA. These changes to the innate immune profile may contribute to the previous observations of improved intracellular stability of 5moU-modified RNA compared to Ψ and m1Ψ.^28^ One possible explanation is that processing or partial degradation of modified RNA or dsRNA species generates 5moU-containing fragments that alter RNA sensor engagement or downstream signaling. This could help to explain why partial 5moU-modification of mRNA is able to evade the immune response. Recent work has demonstrated that certain RNA fragments with 2′-O-ribose modified nucleotides can durably inhibit innate immune sensors TLR7 and TLR8 in a similar manner,^34^ although whether 5moU-containing fragments have comparable activity is not known.

Felgner et al.^18^ previously reported that a 5moU_100_-mRNA vaccine was ineffective at producing an antibody response to the encoded antigen.^18^ In our study, while 5moU_100_ inhibited eGFP mRNA translation *in vitro* and *ex vivo*, when hIgG-encoding mRNA was delivered via LNP, 5moU_100_ performed equally to m1Ψ_100_ *in vivo* (though both were exceeded by 5moU_50_)_._ This may reflect altered entry route, host, and tissue target but corroborates the assertion of Felgner et al. that their 5moU modified mRNA LNP-delivered vaccine was ineffective due to reduced immunogenicity of the mRNA itself. Indeed, here, some of the DEGs in response to m1Ψ (e.g., *PIK3AP1*) are involved in the adaptive immune response. This aligns with the reported benefits of, and considerations in, self-adjuvating mRNAs.^35^^,^^36^ Together, these findings indicate that 5moU may offer advantages over m1Ψ for applications requiring prolonged expression with minimal immune activation, such as gene therapy or monoclonal antibody delivery.

Demonstrating that 25% 5moU can achieve performance comparable to or exceeding that of 100% m1Ψ has practical implications for both functional output and manufacturing efficiency. For example, although 5moU does not alter secondary structure,^26^ modifications that do may benefit even more from stoichiometric tuning. Given that modified nucleotides represent a substantial cost in mRNA production, reducing substitution ratios by up to 4-fold presents a clear economic advantage and enables fine-tuning of mRNA characteristics to cell-type and purpose. More broadly, with over 170 RNA modifications described,^37^ yet few examined across a range of incorporation ratios, our work provides a framework for systematically evaluating how modification stoichiometry shapes RNA behavior. Our work expands this landscape by showing that functional and cell-type-specific responses vary across stoichiometries. As mRNA therapeutics diversify beyond vaccines, the ability to fine-tune molecular properties through partial modification will be central to achieving precise control over expression, tissue targeting, and immune compatibility.

## Methods

### *In vitro* transcription templates

For the eGFP reporter assays, eGFP mRNA was generated from a linearized plasmid template encoding eGFP with minimal 5′ and 3′ UTRs, and a templated poly(A) tail.

For the nanopore sequencing, we modified the pJET vector (Thermo Fisher) via Q5 site-directed mutagenesis (New England Biolabs) to replace the native T7 promoter with the φ2.5 variant, enabling transcription initiation at an adenosine and co-transcriptional capping. A 40 bp adenosine sequence was added downstream for templated poly(A) tailing. Synthesized 5′ UTRs from RPRD2 and ACTB were cloned upstream of firefly or renilla luciferase between the modified promoter and poly(A) sequence. The luciferase templates were generated with PCR using Q5 High Fidelity Polymerase (New England Biolabs), using the forward primer GAAAATTGTCCCAATTAGTA and an OligoDT40 reverse primer and purified using Gel and PCR Clean-up kits (Machery-Nagel).

### *In vitro* transcription reactions

Transcription reactions were carried out according to the T7 transcription reagent manufacturers’ instructions (New England Biolabs), supplemented with 1U/μl murine RNase inhibitor (New England Biolabs). Where required, 0.4 mM CleanCap-AG cap analogue (TriLink Biotechnologies) was included to generate Cap1 mRNA.

IVT reactions were carried out at 37°C, unless indicated otherwise, for 2 h, after which they were incubated for 30 min at 37°C with 2 units of RNase-free DNase (Promega). Sequenced IVTs were purified with Monarch RNA Cleanup Kits (10 μg) (New England Biolabs), eluted in nuclease-free water and quantified with Qubit RNA BR Assay (Thermo Fisher). Transfected IVTs were purified with MEGAClear IVT Purification Kits (Thermo Fisher) and quantified with the Lunatic spectrophotometer using High Lunatic chips and the RNA (Turbidity) setting (Unchained Labs) before quality was checked with TapeStation RNA Screentape (Agilent). IVT templates and conditions are summarized in Table 1.

**Table 1:** Summary of IVT reactions carried out for sequencing and transfection of mRNA

Sequenced mRNA						
Polymerase	Modification	Template	Temp	Cap analogue	rNTP	Chemistry
Promega T7	–	*ACTB*-RLUC	37°C	none	1 mM	RNA002
	–	*ACTB*-FFLUC				
	unmodified	*RPRD2*-RLUC				
	25% 5moU	*RPRD2*-FFLUC				
	–					
NEB HiT7	unmodified	*RPRD2*-RLUC	30°C	none	1 mM	RNA002
	25% 5moU	*RPRD2*-RLUC				
	25% 5moU	*RPRD2*-RLUC	40°C			
		*RPRD2*-RLUC	50°C			
NEB HiScribe	unmodified	*ACTB*-FFLUC (*N* = 3),*RPRD2*-FFLUC (*N* = 3)	37°C	CleanCap-AG	5 mM	RNA004
	25% 5moU	*ACTB*-FFLUC (*N* = 3),*RPRD2*-FFLUC (*N* = 3)				

Transfected mRNA					
Polymerase	Modification	Template	Temp	Cap analogue	rNTP
NEB HiScribe	unmodified (*N* = 3)	eGFP	37°C	CleanCap-AG	5 mM
	25%, 50%, and 100% 5moU (*N* = 3)				
	25%, 50%, and 100% m1Ψ (*N* = 3)				

### Cell culture and transfections

All cells were incubated at 37°C and 5% CO_2_. Media compositions, seeding density, and transfection quantities are listed in Table 2. A549-Dual cells were transfected immediately after seeding; all other cells were transfected a day after seeding at 70%–90% confluence. Transfection was performed using Lipofectamine MessengerMax (Thermo Fisher) according to the manufacturer’s instructions with RNA diluted in Opti-MEM (Thermo Fisher). Transfections of 100% 5moU-modified mCherry mRNA (TriLink Biotechnologies) and unmodified, 5moU-modified, and m1Ψ-modified 300 bp double-stranded RNA (Areterna LLC) were performed in the same way.

**Table 2:** Summary of cell lines and plating conditions used for translational and immunogenic assays of modified mRNA

Cell line	Media	Plate format	Cell number /well	mRNA transfected (ng/well)	dsRNA transfected (ng/well)
A549-Dual	DMEM (Gibco) + 10% FBS (Avantor Seradigm)	96-well	15,000	50	25
A549	DMEM + 10% FBS	24-well	100,000	250	–
BJ fibroblasts	MEM (Gibco) + 10% FBS + 1% NEAA (Gibco)	24-well	100,000	250	–
Hap1	IMDM + 10% FBS	24-well	100,000	250	–
HepG2	MEM + 10% FBS + 1% NEAA	24-well (collagen-coated)	150,000	250	125
Primary myocytes	SkGM®-2 Skeletal Muscle Cell Growth Medium-2 BulletKit	24-well	6,650	500	–
Primary hepatocytes	Williams E Media (WEM) (Sigma), 4% Cocktail B (Hepatocyte Maintenance Pack, Life Technology), 0.001% Dex	48-well	100,000	250	–

### Human primary cells and myoblast differentiation

Human skeletal muscle myoblasts (Lonza) were cultured according to manufacturer’s instructions; briefly, myoblasts were plated at 3,500 cells/cm^2^ and maintained in SkGM-2 Skeletal Muscle Cell Growth Medium-2 BulletKit (CC-3245) and split at a confluency of 80%. For differentiation, cells were plated subconfluently in a collagen-coated 24-well plate, and left to reach a confluency of 80%. Cells were then washed in 1× PBS and media was switched to Skeletal Muscle Differentiation Medium (Promocell). Cells differentiated within 5–7 days.

Cryopreserved plateable human hepatocytes (Lonza) were thawed according to manufacturer’s instructions. Briefly, cells were thawed in a 37°C water bath for <2 min, leaving a small ice crystal, and transferred into 50 mL of pre-warmed plating medium (Williams E Medium supplemented with Cocktail A, FBS, and dexamethasone; Life Technologies CM3000). Cells were centrifuged at 500 ×*g* for 5 min at room temperature (RT), and the supernatant was removed leaving ∼2 mL of media. Cells were gently resuspended and counted using trypan blue exclusion. Hepatocytes were seeded at 50,000–60,000 cells/well in collagen-coated 96-well plates (VWR, 734–0276) pre-wetted with 50 μL plating medium. The cell suspension was added at 100 μL per well and mixed gently to ensure even distribution. Plates were incubated at 37°C, 5% CO_2_ for 4–6 h to allow cell attachment, after which media was replaced with maintenance medium (Williams E Medium supplemented with Cocktail B and dexamethasone; Life Technologies CM4000). Media was refreshed the following morning to remove non-adherent cells, and transfection was performed immediately thereafter.

### Fluorescence translation data acquisition

Fluorescence activity of eGFP mRNA reporter transfection was analyzed using the Incucyte system (2023 VerA). For each cell line except primary myocytes, 4 images were taken per well every 2 h for 24 h. For primary myocytes, 9 images were taken per well to account for the relative size of the cells. Default AI confluence mask settings were used for all analyses except for primary hepatocytes, where classic confluence masks were set using phase minimum areas of 300 μm^2^ and eGFP minimum areas of 100 μm^2^. Data were visualized and statistical analysis was performed in GraphPad prism 10.3.0.

### Fluorescence microscopy

Cells transfected with 100% 5moU-modified mCherry mRNA (TebuBio) were imaged 22 h post-transfection with a DMi8 Inverted Microscope (Leica). Mean fluorescence intensity was calculated using ImageJ 1.54g.^38^ Intensity threshold was adjusted using default parameters.

### A549-Dual cell immune assay

At 20 h after transfection with mRNA, secreted luciferase was quantified by Quanti-Luc reagent. The reagent was resuspended in 25 mL of Milli-Q-purified water as per manufacturer’s recommendations. 20 μL media was aspirated from A549-Dual cells and transferred to a white, flat-bottom 96-well luciferase detection plate (Corning). 50 μL per well of the working Quanti-Luc Reagent was added to the relevant wells of the luciferase detection plate. The luciferase detection plate was then foil-wrapped and tapped repeatedly (but never vortexed) and then allowed to stand for 3–4 min before reading with an Envision 2104 plate reader (PerkinElmer) with a standard 96-well luminescence protocol.

### RT-qPCR of innate-immune activated genes

At 24 h post-transfection, total RNA was isolated by cell lysis with 40 mM DTT-supplemented RLT buffer and purification using the RNeasy Mini spin columns (Qiagen) as directed with on-column DNase digestion. RNA was stored at –80 °C.

cDNA was generated from purified total RNA using the High-Capacity cDNA Reverse Transcription Kit (ThermoFisher) as directed by the manufacturer with 0.75 ng/μL total RNA per reaction. Neat cDNA reaction products were then analyzed with qPCR using the TaqMan Fast Advanced Master Mix (Thermo Fisher). FAM probes for RIG-I (DDH58, Hs01061436_m1), MDA5 (IFIH1, Hs00223420_m1), IFIT1 (Hs01675197_m1), and 18S-rRNA (Hs03003631_g1) were purchased from Thermo Fisher. Liquid handling of probe master mixes into MicroAmp EnduraPlate 384-well plates (Thermo Fisher) was performed with an Echo 650 Acoustic Liquid Handler (Beckman Coulter). qPCR was performed with a QuantStudio7 Real-Time PCR system (Thermofisher) using the Fast advanced PCR protocol over 40 cycles and 2 μL reaction volumes. Relative quantification (RQ) values were calculated using 18S rRNA as an endogenous control.

### Illumina sequencing

For each treatment, 300 ng of total RNA were submitted to Source Bioscience (Cambridge) for sequencing. There, samples were poly(A) selected before cDNA generation with the NEB Ultra II DNA Library Preparation Kit (New England Biolabs), targeting 40,000,000 paired end reads of 150 bp lengths. The sequencing was performed on the Illumina NovaSeq X instrument using 25B flow cells, with a read configuration of 151-10-10-151 (150 bp paired-end sequencing). Four experimental conditions were tested, m1Ψ_100_, 5moU_25_, unmodified mRNA, and mock, in triplicate. These were performed in two separate batches of sequencing, one batch containing mock and m1Ψ_100_, and a separate batch comprising mock, 5moU_25_, and unmodified mRNA treatments; all were performed in triplicate.

### Differential gene expression analysis

Raw reads were trimmed and aligned to the human genome (GRCh38) with STAR, using default parameters but specifying a .gtf file, gene counting was performed with HTseq-count specifying -r pos and -s reverse, but otherwise default parameters, and readcounts imported into R v4.3 via tximport 1.32.0. Downstream analysis was performed in DESeq2 1.44.0 with default parameters using the model ∼ modification, where the modification was defined in the sampleTable as mock, m1Ψ, or 5moU. Mocks were specified as the baseline for all pairwise comparisons. For sample-by-sample Pearson correlation matrix (Figure S7) rlog transformation (blind = TRUE) was applied to the DESeq2 dataset. Similarly, for gene-panel heatmaps, rlog transformation (blind = TRUE) was applied, followed by row-wise Z-scoring. The DESeq2 package applied Wald tests for pairwise contrasts, and multiple testing correction was performed using the Benjamini-Hochberg method (false discovery rate [FDR] < 0.05). Log_2_ fold-change shrinkage was applied using apeglm 1.26.1. Functional annotation and pathway analysis were conducted on significantly upregulated genes (absolute log_2_FoldChange > 1, adjusted *p* < 0.01). GO enrichment was performed with clusterProfiler 4.12.6, and Reactome-based GSEA used fgsea 1.30.0, ranking all detected genes in each comparison by Wald statistic (log_2_ fold change divided by its standard error), to sort genes by both magnitude and variability. For gene counting in supplemental figures and tables, custom R code deployed org.Hs.eg.db and reactome.db to download lists of genes in specific GO biological processes or Reactome datasets and then count the occurrences of those genes appearing in the DESeq2 outputs at the thresholds specified in the figure legends. A single mock-treated replicate was excluded from some analyses in supplemental data. Detailed analysis of the sample removal and its effect on the data are described in Figure S7. Briefly, mock-treated replicate 2904_1 exhibited higher immune gene activation than the other mock-treated samples. This may reflect biological or technical variation. Inclusion of 2904_1 did not reduce the sensitivity of differential gene expression analysis when comparing mock-treated samples with unmodified or m1Ψ-modified RNA treatment. However, exclusion of 2904_1 from the 5moU analyses resulted in the identification of additional DEGs, as shown in Figures S7 and S8.

Data visualization employed ggplot2 v4.0.0, data.table v1.17.8, cowplot v1.2.0, and patchwork v1.3.2 for figure assembly. Principal component analysis (PCA) and sample correlation analysis were generated using DESeq2 and pheatmap. Volcano plots, enrichment bubble plots, and comparative scatterplots of effect sizes were generated from DESeq2 outputs.

For analysis of basal expression of innate immune sensors, basal expression data were obtained from the Human Protein Atlas^32^ rna_celline.tsv file (64 cell lines, consensus normalised expression (NX) values), and custom R code was used to perform the normalization of each gene within a defined set of cell lines. For each gene, NX values were normalized so that the highest expressing cell line of those defined was assigned a value of 100, and all others expressed relative to this maximum. These normalized values were plotted as horizontal dot plots using ggplot2 to compare the basal expression of specific genes across the human protein atlas.

Figures 4F and 4G were generated in GraphPad prism 10.3.0. Full heatmaps with individually labeled genes are in Figure S8 and differential expression analysis is in Table S5.

### *In vivo* studies with mRNA

#### hIgG mRNA synthesis

Two separate mRNA transcripts were prepared for the IgG HC and LC. Each mRNA transcript comprised a 5′ cap structure incorporated through the inclusion of a cap analogue during mRNA synthesis by a T7 polymerase-driven IVT reaction; a 5′ UTR sequence from the human hydroxysteroid 17-beta dehydrogenase 4 gene; a signal sequence from the human kappa Ab LC; an open reading frame (respectively coding for IgG HCs or LCs); a 3′ UTR sequence taken from the human albumin gene; a defined polyA_80_ tail. Uridine was replaced with either 50% or 100% 5moU or m1Ψ in the above IVT reaction mixes. The mRNA vectors were prepared from corresponding DNA plasmids containing the entire mRNA cassettes as described above, along with a T7 polymerase promoter sequence upstream of the mRNA cassette to allow synthesis of mRNA using IVT. The DNA template was linearized by a restriction digestion before IVT, to ensure all mRNA molecules terminate directly after the poly(A) tail.

#### LNP formulation of mRNA

LNPs were prepared by microfluidic mixing as previously described.^39^ Briefly, lipid components dissolved in ethanol were combined with mRNA in RNase-free citrate buffer (50 mM, pH 5) at a 1:3 (v/v) ratio, using a total flow rate of 12 mL/min on a NanoAssemblr system (Cytiva). Immediately following mixing, the LNPs were dialyzed overnight against PBS. The LNP formulation encapsulating mRNA consisted of MC3 ionizable lipid, cholesterol, DSPC, and DMG-PEG at a molar ratio of 50:38.5:10:1.5, respectively, with a total lipid-to-mRNA mass ratio of 20:1 (corresponding to an N:P ratio of 6). Particle size and polydispersity were assessed by dynamic light scattering in the Zetasizer Nano ZS (Malvern Instruments). Two mRNAs encoding HC and LC of the IgG were co-encapsulated at a 1:1 molar ration. mRNA encapsulation efficiency and concentration were quantified using the RiboGreen assay (Invitrogen) according to the manufacturer’s instructions. All formulations were freshly prepared, stored at 2°C–8°C, and used within three days. IgG mRNA was encapsulated.

### Mouse injections with mRNA/LNP

BALB/c mice were injected intravenously in the tail vein with 200 μL mRNA/LNP formulation encoding hIgG diluted in PBS. After 48 h mice were bled via the sub-mandibular route and sera evaluated for antibody concentration by ELISA.

All animal studies were approved by the AstraZeneca Institutional Animal Care and Use Committee and conducted in an AAALAC-accredited facility in compliance with the US regulations governing the housing and use of animals. Male and female BALB/c mice were obtained from The Jackson Laboratory.

### Human IgG quantification in mouse sera by ELISA

Maxisorp plates (Nunc) were coated overnight at 4°C with 100 μL of goat anti-human IgG (Invitrogen) diluted at 1 mg/mL in 0.2 M carbonate/bicarbonate. Three washes with PBS 0.1 % were performed between each incubation. Plates were blocked with PBS 5% BSA for 1.5 h at RT. Purified IgG standards, quality control (QC) samples, and sera samples (pre-diluted in mouse sera) were prepared in assay buffer with a 1:100 minimum required dilution and incubated for 1.5 h at RT on a 200 rpm shaking platform. The IgG bound was detected with 100 μL horseradish peroxidase-conjugated goat anti-human IgG (Invitrogen) at 1:20,000 dilution in assay buffer. After 0.5-h incubation at RT, 100 μL 3,3′,5,5′-tetramethylbenzidine (TMB) was added, and the reaction was stopped with 100 μL H_2_SO_4_. The OD_450_ was measured with a spectrophotometer. The standard curve, comprising 11 concentrations, was generated using a 4-parameter logistic curve fit [y = ((A-D)/(1+(x/C)ˆB)) + D] without weighting in SoftMax Pro (SMP) software, v5.4.5 The concentration of human antibody in serum samples was determined relative to the standard curve.

### Thin layer chromatography and densitometry

Thin layer chromatography (TLC) was performed as described previously.^40^^,^^41^^,^^42^ First, 100 ng of RNA was digested with 1000 units of T1 Nuclease (Invitrogen) in PNK Buffer (New England Biolabs) and incubated at 37 °C for 30 min. The 5′-OH ends of RNA fragments were then radiolabeled with 5 μCi 3000Ci/mmol γ-^32^P ATP (Revvity) and 10 units of T4 PNK (NEB) for 3 h at 37 °C. This reaction was then precipitated with 3 volumes of ethanol and 0.1 volume of 3M sodium acetate, pelleted, and resuspended in nuclease-free water. Radiolabeled RNA was digested with 100 units of nuclease P1 (New England Biolabs), before spotting on a 20 cm × 20 cm Cellulose-F glass plate (Merck). Loaded (spotted) TLC plates were run in two dimensions. We used published buffer recipes^42^^,^^43^ A then B, or buffer A then C. Buffer A consisted of 5:3 v/v isobutyric acid: 0.5 M NH_4_OH. Buffer B consisted of 70:15:15 v/v/v isopropanol:HCl:H_2_O. Buffer C consisted of 100:60:2 v/w/v sodium phosphate, 0.1 M (pH 6.8), ammonium sulfate, n-propanol. TLC plates were imaged with phosphor imaging and the FX Phosphor imager (Bio-Rad Laboratories) or Sapphire FL Biomolecular imager (Azure Biosystems) and densitometry using QuantityOne v4.6.8 (Figure S2).

### Oxford nanopore sequencing library preparation

RNA002 libraries were prepared with the SQK-RNA002 kit (Oxford Nanopore Technologies) according to the manufacturer’s protocol, and sequencing was performed on Flongle R9.4.1 Flow Cells. The volume of RTA and RMX reagents used were one-sixth of that in the manufacturer’s protocol (accounting for the size difference between MinIon and Flongle flow cells). cDNA was generated with Induro Reverse Transcriptase (New England Biolabs). For runs with mixed transcripts, up to 100 fmol of each species was loaded and run for >16 h.

RNA004 libraries were prepared with the SQK-RNA004 kit (Oxford Nanopore Technologies) and sequencing performed with the MinION RNA004 Flow cells. Half volumes of RTA and RMX reagents in the written protocols were used. For each run, 100 fmol of each transcript was prepared to a total of 150 ng in mixed runs. Specific templates and their reaction conditions are described in Table S1.

### Oxford nanopore data preparation and analysis

Per-base mismatch, insertion, deletion, and quality metrics were derived using *EpiNano_Variants.py* from the EpiNano package.^31^ For RNA002, fast5 files from individual runs were pooled and split into fast5 files of equal read numbers. fast5 files from repeated runs were pooled and split into two fast5 files of equal read numbers. Basecalling for RNA002 fast5 files was performed with Guppy v2.24-r1122 and basecalling model rna_r9.4.1_70bps_hac. Genes from the same run were separated by filtering base called fastq files with nanoq v0.8.2 by quality (minimum cutoff 5) and transcript length (Table S3) (-q 5 -L 1000/1700/1400/2200 -I {input_fastq} -o {output_fastq}). These filtered fastq files were then aligned to the reference gene with minmap2 v2.17-r914 (-a -uf -ax splice -k14). For a run containing 2 transcripts, this produced 2 fasta files, which each provided a fastq file and bam alignment file for each gene.

Separated fasta, fastq, and bam files were used for signal-based analysis. Nanopolish v0.14.0 was used to index fastq and fast5 files (-s {run_sequencing_summary.txt} -d {fast5_directory} {fastq_directory}) and eventalign fastqs with bam files (--reads {fastq_directory} --bam {bam} --genome {reference.fasta} --print-read-names --scale-events --samples > {eventalign_1.tsv}. Eventalign .tsvs were collapsed with nanocompore v1.0.4 (-t 3 -I {evenalign.tsv} -o {eventalign_collapse_directory} before sample comparison (sampcomp --file_list1 ./unmod_eventalign_collapse1.tsv,./ unmod_eventalign_collapse2.tsv --file_list2 ./ mod_eventalign_collapse1.tsv,./nmod_eventalign_collapse2.tsv --label1 unmod --label2 mod --fasta {reference.fasta} --outpath {output_directory}. Aligned bam files were pooled by gene from each run and sequence-level analysis performed with Epinano version 1.2.0 (Epinano_Varients.py -b {.bam} -r {reference.fasta} -o {directory}).

RNA004 basecalling was performed with Dorado 0.9.0 with the basecalling model rna004_130bps_hac@v5.0.0 (guppy_basecaller --emit-fastq -i {pod5_directory} -s. Basecalled fastq files were filtered by quality (cutoff = 5) and length for each target gene, producing two files for mixed runs. ACTB-FFLUC and RPRD2-FFLUC transcripts were separated from nanoq filtered fastq files by alignment with minimap2 v2.29-r1283 (-a -uf -ax splice -k14) first against the different 5′ UTRs, and then against their full-length gene. bam files for each replicate of each gene, either unmodified or 25% 5moU modified, were subject to sequence-level modification detection with Epinano v1.2.0 as before. The Epinano files for each replicate were averaged across gene and modification condition.

Numbers of reads, filtered reads, and mean Q scores are detailed in Table S2; lengths cutoffs used are detailed in Table S3. Plots were made with R v4.4.1 using ggplot2 4.0.0.

### DRS statistics

Per-site comma separated variable (CSV) outputs from EpiNano v1.2.0 were imported into R v4.3 for analysis. Data processing was performed using data.table v1.17.8, and plots were generated with ggplot2. Per-base mismatch, deletion, insertion, and quality metrics were compared to generate an error profile in each replicate (Figure S3). Visual inspection of boxplots identified mismatch frequency as the most informative indicator, consistent across replicates. To confirm this trend, mismatch frequencies were plotted across base positions after subsetting both modified and unmodified datasets to defined nucleotide ranges, with the reference base on the *x* axis. This visualized modification-associated differences in mismatch rate over short, fixed sequence windows, revealing elevated mismatch in U-containing kmers (RNA002), which became more specific in RNA004.

Δ-mismatch values were obtained by subtracting per-base mismatch frequencies of the unmodified dataset from those of the 25% 5moU dataset, and plotted along transcript coordinates to identify regions showing increased mismatch associated with modification.

For quantification across broader regions, counts of mismatched (“mis”) and matched (“match”) reads were summed in 150-nt bins for each replicate and condition. A binomial generalized linear model (GLM) was fitted using stats, with mis/match as the response and condition × bin + replicate as predictors, treating replicate as a blocking factor. Log_2_ odds ratios (modified – unmodified) and 95% confidence intervals were estimated with emmeans v1.11.2.8, and Benjamini-Hochberg correction was applied to control the FDR. Significant bins (FDR < 0.05) were highlighted in orange.

Pairwise bin-to-bin contrasts of the GLM effect (mod – unmod) were used to construct correlation heatmaps, with –log_10_-transformed adjusted *p* values indicating bin-level concordance in modification-associated mismatch enrichment. Heatmaps and figure layouts were assembled with ggplot2 v4.0.0, cowplot v1.2.0, and patchwork v1.3.2.

### Dot blot analysis

Dot blots for dsRNA detection were performed largely as previously described.^44^ In brief, samples were ethanol precipitated and resuspended in nuclease-free water. Unmodified, 5moU25, 5moU100, m1Ψ25, and m1Ψ100 eGFP RNAs, together with an ssRNA ladder (NEB), were prepared at 1 μg/μL. A dsRNA ladder (NEB) was prepared at 500 ng/μL to avoid signal saturation. For each sample, 1 μL was spotted onto a Biodyne B positively charged nylon membrane (Thermo Fisher Scientific) and allowed to air dry. Membranes were UV crosslinked at 254 nm (120 mJ/cm^2^), washed briefly in TBST, and blocked overnight in 5% BSA in TBST at 4°C with agitation. Membranes were washed in TBST before incubation with J2 anti-dsRNA antibody (Jena Bioscience) diluted 1:5000 in 1% BSA-TBST. Following three washes in TBST, membranes were incubated with HRP-conjugated anti-mouse antibody (Abcam) in 1% BSA-TBST. After final washes in TBST, chemiluminescent signal was detected using SuperSignal West Pico Chemiluminescent Substrate (Thermo Fisher Scientific) on a ChemiDoc imaging system (Bio-Rad). Signal was quantified using Quantity One software (Bio-Rad). Statistical significance between sample groups was assessed using a Kruskal-Wallis test with Dunn’s multiple comparisons test for post hoc pairwise comparisons.

## Data and code availability

RNA sequencing data is available on Gene Expression Omnibus GSE312827; basecalled DRS data is available on Gene expression omnibus GSE313502 (luciferase mRNA) and GSE335071 (eGFP mRNA), data underlying figures found in supplemental data; computer code and raw nanopore read/signal data available upon reasonable request.

## Acknowledgments

The authors thank the colleagues at the University of Nottingham and the members of the AstraZeneca RNA Therapy team for valuable discussions and critical feedback throughout this work. J.R.M. and S. Masterson. were supported by 10.13039/501100000268BBSRC DTP CASE studentships (BB/T008369/1), sponsored by 10.13039/100004325AstraZeneca. N.A. received funding from the 10.13039/501100000288Royal Society (RG\R2\232181), and E.B. received funding from the Leverhulme Trust (RPG-2020-284). Figure schematics in the graphical abstract and Figures 3E, S1C, and S1D were created in BioRender.

## Author contributions

Conceptualization, N.A., J.B., G.T., R.G.F., and J.M.D.; methodology, N.A., J.B., G.T., R.G.F., J.R.M., V.J., N.P.M., C.J.H., and M.L.; investigation, J.R.M., S. Masterson, J.B., R.R., S. Marelli, E.B., and L.S.; resources, N.A., V.J., R.G.F., and E.B.; data curation; N.A., J.R.M., L.G., C.H., F.F., N.P.M., and M.L.; formal analysis, N.A., J.R.M., L.G., C.H., F.F., N.P.M., and M.L.; visualization, N.A., J.R.M., and M.L.; supervision, N.A., R.G.F., J.B., G.T., V.J., N.P.M., C.J.H., and J.M.D.; writing – original draft, J.R.M., N.A., R.G.F., and J.M.D.; writing – review and editing, all authors; project administration, N.A., J.B., and G.T.; validation, J.R.M., E.B., S. Masterson, and N.A.; experimental design, N.A., J.B., G.T., R.G.F., J.R.M., V.J., N.P.M., C.J.H., and M.L.

## Declaration of interests

L.G., C.H., F.F., R.R., S. Marelli, J.B., and G.T. are the employees of AstraZeneca PLC. J.R.M. and S. Masterson are BBSRC-DTP CASE students sponsored by AstraZeneca PLC. AstraZeneca employees acted as project supervisors and contributed to the study design and interpretation of results.

## Supplemental information

Document S1. Figures S1–S9 and Tables S1–S3

Table S4. Ct and calculated RQ values from qPCR measurement of RIGI, MDA5, and IFIT1 expression

Table S5. Differential gene expression and enrichment data underpinning Figure 4

Document S2. Article plus supplemental information
