# Supplementary material for: Sub-stoichiometric 5-methoxyuridine modification enables tunable immune evasion and protein expression from synthetic mRNAs
Source: Mol Ther Nucleic Acids. 2026 Aug 12;37(3):103058. doi: 10.1016/j.omtn.2026.103058 (PMC13543807; doi:10.1016/j.omtn.2026.103058)
Supplement: Supplementary file 1 [file mmc1.pdf]

OMTN, Volume *37*

Supplemental information

Sub-stoichiometric 5-methoxyuridine modification enables tunable immune evasion and protein expression from synthetic mRNAs

Jonathan R. Miles, Sarah Masterson, Eleanor Bellows, Fabio Fisher, Ritu Rani, Sara Marelli, Colin Hardman, Luigi Grassi, Luis Santos, Victoria James, Nigel P. Mongan, Christopher J. Hayes, Matthew Loose, Janet M. Daly, James Button, George Thom, Rupert G. Fray, and Nathan Archer

Table S1 – Summary of outputs of sequencing runs performed with SQK-RNA002 and Flongle R9.4.1 flow cells

Run	IVT	Polymerase	Modification	Total Reads (k)	Processed Output	Filtered Reads	Mean Q Score
1	*ACTB*-RLUC<br>*ACTB*-FFLUC	Promega WT	Unmodified	102.27	ACTB-RLUC Unmodified	68993	11
					ACTB-RLUC 25% 5moU	27801	9.9
2	*ACTB*-RLUC<br>*ACTB*-FFLUC		25% 5moU	77.92	ACTB-FFLUC Unmodified	13685	11.2
					ACTB-FFLUC 25% 5moU	10614	9.8
3&4	*RPRD2*-RLUC<br>*RPRD2*-FFLUC		Unmodified	17.91	RPRD2-RLUC Unmodified	28281	10.7
			Unmodified	93.63	RPRD2-RLUC 25% 5moU	30545	10.2
5&6	*RPRD2*-RLUC<br>*RPRD2*-FFLUC		25% 5moU	23.08	RPRD2-FFLUC Unmodified	7511	11
			25% 5moU	93.63	RPRD2-FFLUC 25% 5moU	6536	10.6
7	*RPRD2*-RLUC	NEB HiT7	30 °C Unmodified	23.98	30 °C Unmodified	18542	10.9
8&9			30 °C 25% 5moU	8.92<br>28.19	30 °C 25% 5moU	4750	8.7
10			40 °C 25% 5moU	6.3	40 °C 25% 5moU	3805	9.9
11			50 °C 25% 5moU	28	50 °C 25% 5moU	3988	9.1

Table S2 – Summary of outputs of sequencing runs performed with SQK-RNA004 and MinION RNA flow cells

<table>
<tr><th>Run</th><th>IVT</th><th>Modification</th><th>Total Reads (k)</th><th>Processed Output</th><th>Filtered Reads</th><th>Mean Q Score</th></tr>
<tr><td>1</td><td>ACTB-FFLUC rep1</td><td>Unmodified</td><td>406.12</td><td>ACTB-FFLUC Unmodified 1</td><td>88073</td><td>15.1</td></tr>
<tr><td>2</td><td>ACTB-FFLUC rep1</td><td>25% 5moU</td><td>252.12</td><td>ACTB-FFLUC Unmodified 2</td><td>171535</td><td>16.5</td></tr>
<tr><td rowspan="2">3</td><td>ACTB-FFLUC rep2</td><td rowspan="2">Unmodified</td><td rowspan="2">425.94</td><td rowspan="2">ACTB-FFLUC Unmodified 3</td><td rowspan="2">200,000</td><td rowspan="2">17.9</td></tr>
<tr><td>RPRD2-FFLUC rep1</td></tr>
<tr><td rowspan="2">4</td><td>ACTB-FFLUC rep 2</td><td rowspan="2">25% 5moU</td><td rowspan="2">407.69</td><td>ACTB-FFLUC 25% 5moU 1</td><td>105520</td><td>11.5</td></tr>
<tr><td>RPRD2-FFLUC rep1</td><td>ACTB-FFLUC 25% 5moU 2</td><td>190939</td><td>10.1</td></tr>
<tr><td rowspan="2">5</td><td>ACTB-FFLUC rep3</td><td rowspan="2">Unmodified</td><td rowspan="2">425.98</td><td rowspan="2">ACTB-FFLUC 3</td><td rowspan="2">200,000</td><td rowspan="2">11</td></tr>
<tr><td>RPRD2-FFLUC rep2</td></tr>
<tr><td rowspan="2">6</td><td>ACTB-FFLUC rep3</td><td rowspan="2">25% 5moU</td><td rowspan="2">423.71</td><td>RPRD2-FFLUC Unmodified 1</td><td>11668</td><td>16.2</td></tr>
<tr><td>RPRD2-FFLUC rep2</td><td>RPRD2-FFLUC Unmodified 2</td><td>18070</td><td>17.4</td></tr>
<tr><td rowspan="2">7</td><td rowspan="2">RPRD2-FFLUC rep3</td><td rowspan="2">Unmodified</td><td rowspan="2">203.72</td><td>RPRD2-FFLUC Unmodified 3</td><td>32758</td><td>16.2</td></tr>
<tr><td>RPRD2-FFLUC 25% 5moU 1</td><td>11751</td><td>10.5</td></tr>
<tr><td rowspan="2">8</td><td rowspan="2">RPRD2-FFLUC rep3</td><td rowspan="2">25% 5moU</td><td rowspan="2">224.8</td><td>RPRD2-FFLUC 25% 5moU 2</td><td>11786</td><td>10.8</td></tr>
<tr><td>RPRD2-FFLUC 25% 5moU 3</td><td>27598</td><td>10</td></tr>
<tr><td>9</td><td>eGFP</td><td>Unmodified</td><td>80.6</td><td>GFP.U_1</td><td>60960</td><td>24.1</td></tr>
<tr><td>10</td><td>eGFP</td><td>Unmodified</td><td>214.3</td><td>GFP.U_2</td><td>168850</td><td>26.4</td></tr>
<tr><td>11</td><td>eGFP</td><td>Unmodified</td><td>196.5</td><td>GFP.U_3</td><td>160772</td><td>28.2</td></tr>
<tr><td>12</td><td>eGFP</td><td>25% 5moU</td><td>128.3</td><td>GFP.5moU.1</td><td>107901</td><td>23.8</td></tr>
<tr><td>13</td><td>eGFP</td><td>25% 5moU</td><td>801.5</td><td>GFP.5moU.2</td><td>630003</td><td>23.3</td></tr>
<tr><td>14</td><td>eGFP</td><td>25% 5moU</td><td>186.6</td><td>GFP.5moU.3</td><td>139725</td><td>23.2</td></tr>
</table>

Table S3 - Summary of sequenced transcript lengths and length cutoffs used for quality control

Construct	Transcript length (nucleotides)	Minimum length cutoff (nucleotides)
ACTB Renilla	1210	1000
ACTB Firefly	1945	1700
RPRD2 Renilla	1701	1400
RPRD2 Firefly	2433	2200
eGFP	910	600

Table S4.xlsx Table S4 workbook containing. Ct and calculated RQ values from qPCR measurement of RIGI, MDA5, and IFIT1 expression, with 18S-rRNA as an internal control. Table containing values measured after treatment of HepG2, A549, Hap1 and BJ Fibroblast cell lines after treatment with differentially modified reporter mRNA, dsRNA constructs, or mock lipofectamine control. Each cell line treatment performed with N=3 biological replicates, and each qPCR measurement of target genes performed with N=3 technical replicates.

Table S5.xslx. Table S5 Workbook containing all differential gene expression and enrichment data completed during this work (Figure 4). Sheets are named for the comparisons and include:

5moU.vs.Mock Differential gene expression analysis output of cells responding to $5moU_{25}$ modified mRNA.

unmodified.vs.Mock Differential gene expression analysis output of cells responding to unmodified mRNA.

5moU.vs.Mock.2904_1 removed Differential gene expression analysis output of cells responding to $5moU_{25}$ modified mRNA, after mock 2904_1 is removed.

m1psu.vs.Mock Differential gene expression analysis output of cells responding to $m1\Psi_{100}$ modified mRNA.

Unmodified GO Down and Unmodified GO Up – Gene ontology, biological process enrichment on down or upregulated differentially expressed genes (DEGs) in response to unmodified RNA (compared to mock).

Unmodified REACT Down and Unmodified REACT Up – Reactome enrichment on down or upregulated DEGs in response to unmodified RNA (compared to mock).

GSEA GO Unmodified, GSEA GO m1psU, GSEA GO 5moU - Gene set enrichment analysis for gene ontology, biological process terms performed on all genes detected during differential gene expression analysis on cells responding to Unmodified, 25% 5moU-modified, or 100% m1PsU-modified (m1Ψ) compared to mock, ranked by wald statistic.

GSEA REACT Unmodified, GSEA REACT m1psU, GSEA REACT 5moU, - Gene set enrichment analysis for Reactome terms performed on all genes detected during differential gene expression analysis on cells responding to Unmodified, 25% 5moU-modified, or 100% m1PsU-modified (m1Ψ) compared to mock, ranked by Wald statistic.

GSEA REACT 5moU Outlier Removed and GSEA GO 5moU Outlier Removed – Gene set enrichment analysis for Reactome and gene ontology biological process terms on cells responding to 25% 5moU-modified, compared to mock with the outlier mock removed.

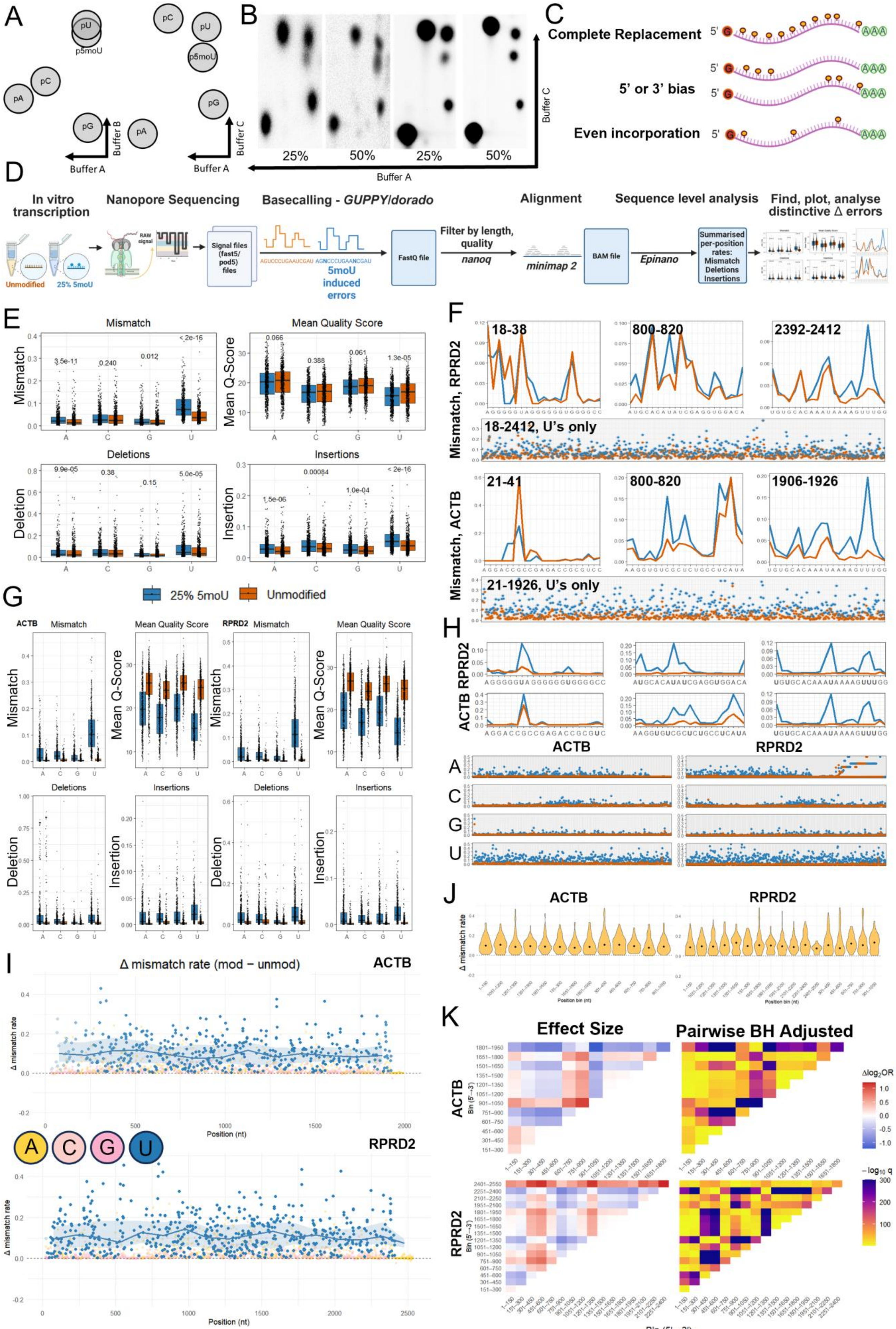

A
pU
p5moU
pC
pA
pG
Buffer B
Buffer A
pC
pU
p5moU
pG
pA
Buffer C
Buffer A
B
25%
50%
25%
50%
Buffer C
Buffer A
C
Complete Replacement
5' or 3' bias
Even incorporation
D
In vitro transcription
Nanopore Sequencing
Basecalling - GUPPY/dorado
Alignment
Sequence level analysis
Find, plot, analyse distinctive Δ errors
Unmodified
25% 5moU
Signal files (fast5/ pod5) files
5moU induced errors
FastQ file
Filter by length, quality
nanoq
minimap 2
BAM file
Epinano
Summarised per-position rates: Mismatch Deletions Insertions
E
Mismatch
Mean Quality Score
Mean Q-Score
Deletions
Deletion
Insertions
Insertion
F
18-38
800-820
2392-2412
Mismatch, RPRD2
18-2412, U's only
21-41
800-820
1906-1926
Mismatch, ACTB
21-1926, U's only
G
25% 5moU
Unmodified
ACTB
RPRD2
H
ACTB
RPRD2
I
Δ mismatch rate (mod − unmod)
ACTB
Δ mismatch rate
Position (nt)
RPRD2
J
ACTB
RPRD2
Δ mismatch rate
Position bin (nt)
K
Effect Size
Pairwise BH Adjusted
Bin (5'→3')
Δlog2OR
−log10 q

Figure S1. Nanopore direct-RNA sequencing based quality control of substoichiometric modified mRNAs (A) Schematic of the migration of nucleotides A, C, G, U and 5moU in two-dimensional thin layer chromatography with buffer B or C[25]. (B) 2D-TLC with the buffer A-C combination on $5moU_{25}$ and $5moU_{50}$ IVT mRNA, two replicates of each shown. (C) Schematic showing potential incorporation trends. (D) Schematic showing Nanopore sequencing based, nucleotide agnostic pipeline based on Epinano. (E) Boxplots for mismatch, mean quality score, deletions, and insertion metrics averaged for each nucleotide within the RPRD2 sequencing run from RNA002 chemistry, p-values shown were calculated by Wilcoxon signed rank test. (F) Example 20-nt windows (positions shown) showing mismatch rates for unmodified (red) and modified RNA (blue) for both RPRD2 and ACTB transcripts. Mismatch rate plotted for all uracils across the transcript (scatter). (G) Boxplots describing the error profiles for RNA004 chemistry for ACTB and RPRD2 IVT mRNAs. (N=3). (H) Mismatch plotted for RNA004 chemistry across the 20-nt windows, and plotted for each nucleotide across the full lengths of the RNA transcript. (I) Δ-mismatch (mismatch in modified – mismatch in unmodified) plots ribbons showing interquartile range along the transcript. (J) Δ-mismatch averaged per 150-nt bin and shown as violin plots. (K) Heatmap of GLM results (binomial model with logit link) comparing mismatch vs match counts as a function of condition, bin, and their interaction, with replicate as a blocking factor. For effect size and Benjamini Hochberg adjusted p-values.

Reaction 50% 5moU

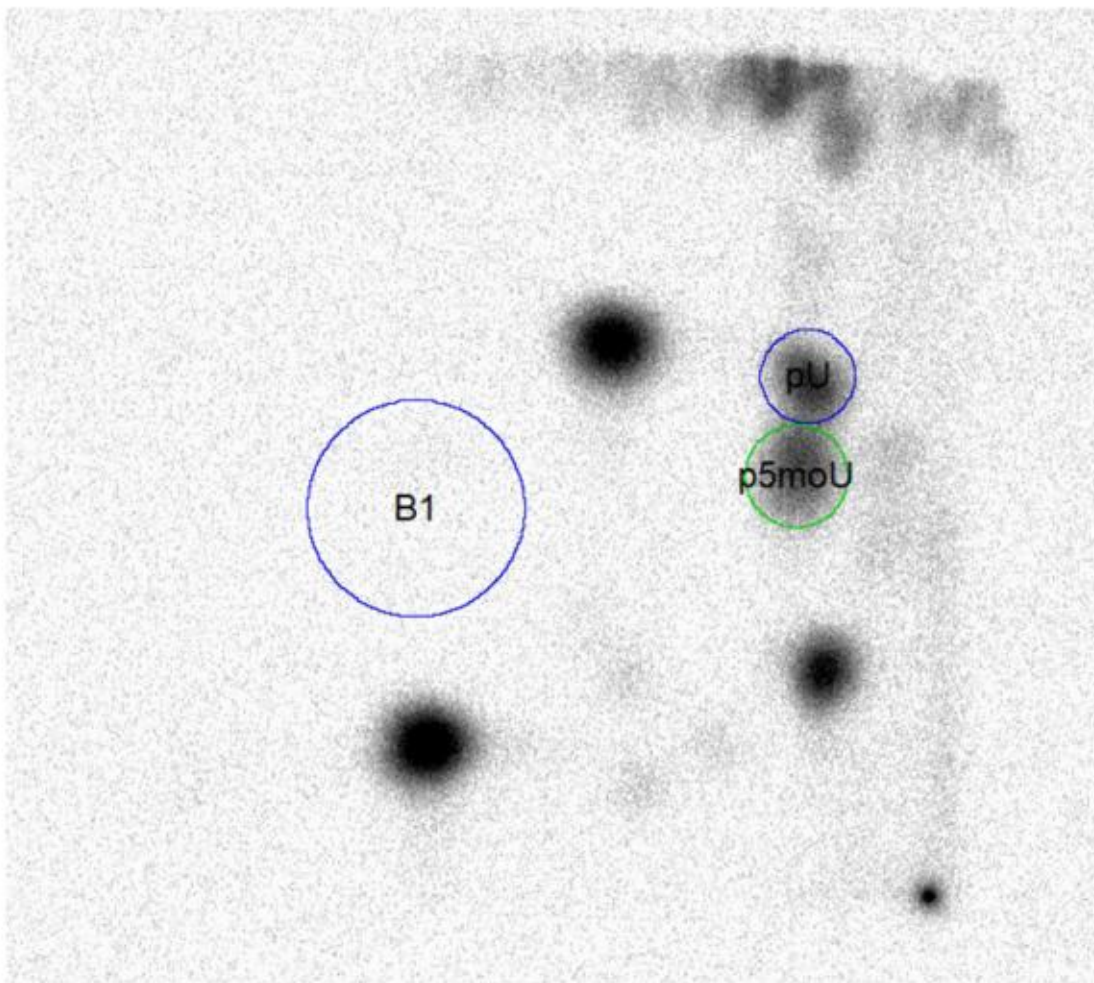

Index	Name	Volume CNT*mm2	Adj. Vol. CNT*mm2	% Adj. Vol.	Concentration	Density CNT/mm2
1	p5moU	3299.980098	3235.798083	49.99 ←	N/A	2894.465312
2	pU	3290.500098	3236.473714	50.01	N/A	3428.675526
3	B1	286.4800085	0.000000000	N/A	N/A	56.29507191

Reaction 25% 5moU

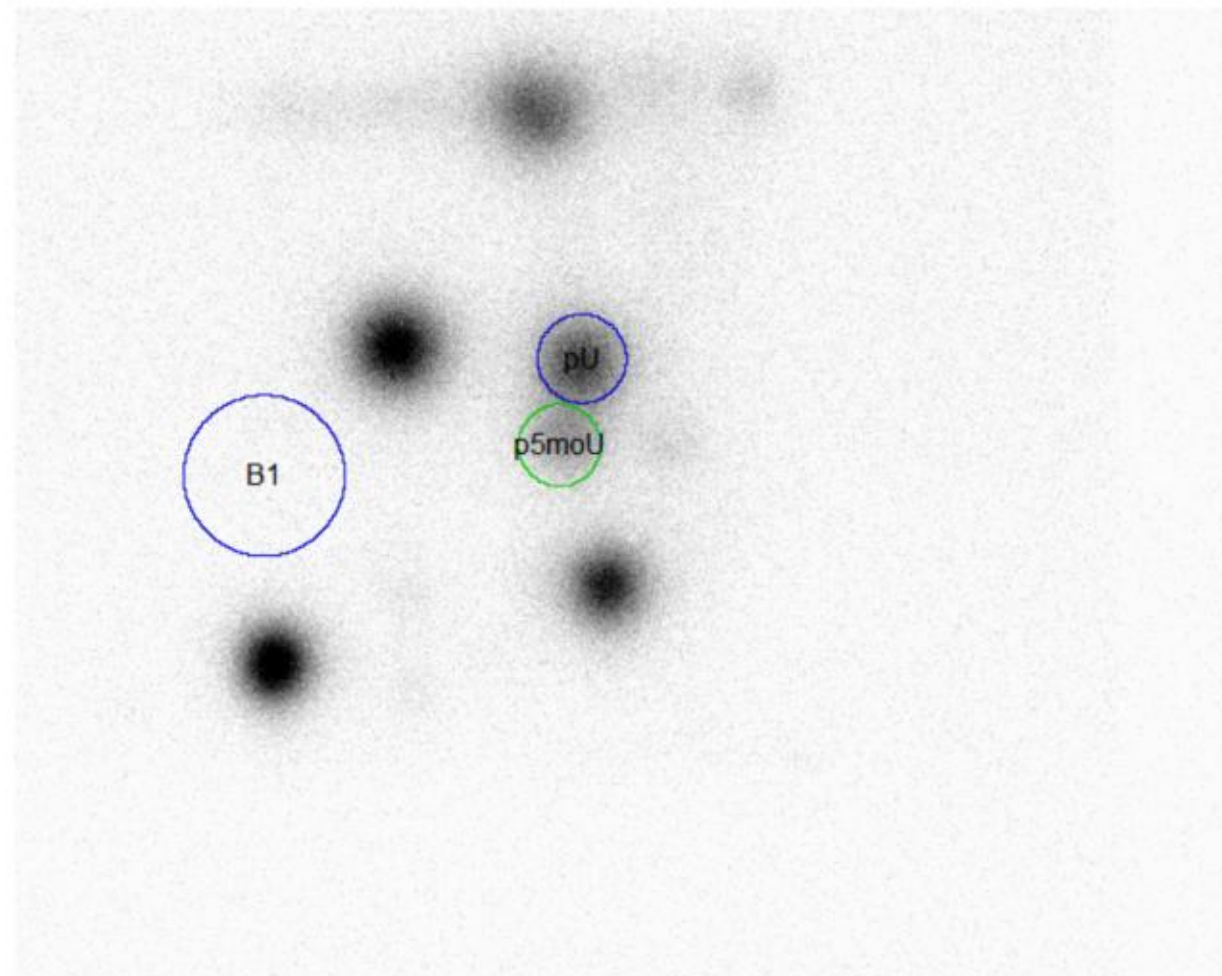

Index	Name	Volume CNT*mm2	Adj. Vol. CNT*mm2	% Adj. Vol.	Concentration	Density CNT/mm2
1	pU	11054.63115	10840.87026	73.25	N/A	8521.258650
2	p5moU	4141.060432	3959.397448	26.75 ←	N/A	3756.063100
3	B1	699.2500729	0.000000000	N/A	N/A	164.7736473

Reaction 50% 5moU

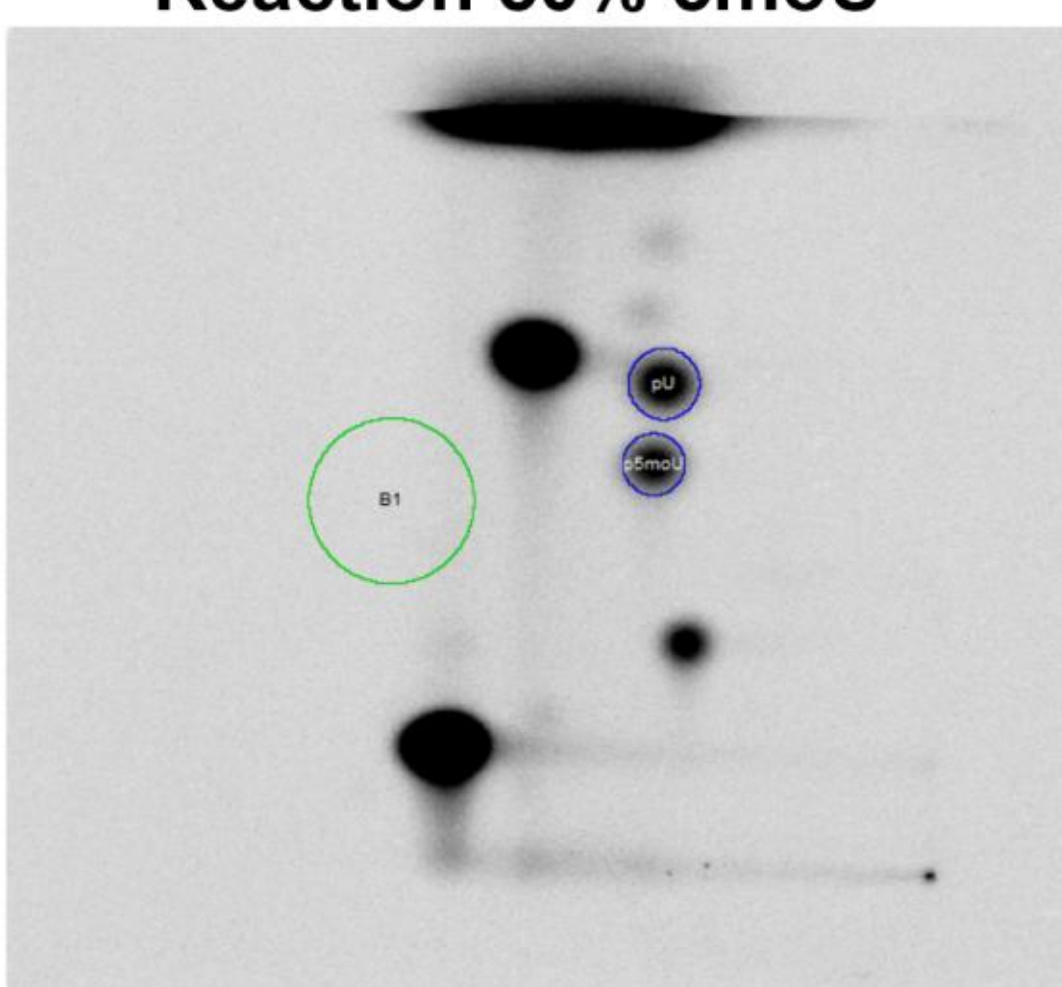

Index	Name	Volume INT*mm2	Adj. Vol. INT*mm2	% Adj. Vol.	Concentration	Density INT/mm2
1	p5moU	393312.4890	368600.6260	40.34 ←	N/A	102218.8708
2	pU	577678.3861	545195.1990	59.66	N/A	114215.8337
3	B1	174844.6385	0.000000000	N/A	N/A	6422.421862

Reaction 25% 5moU

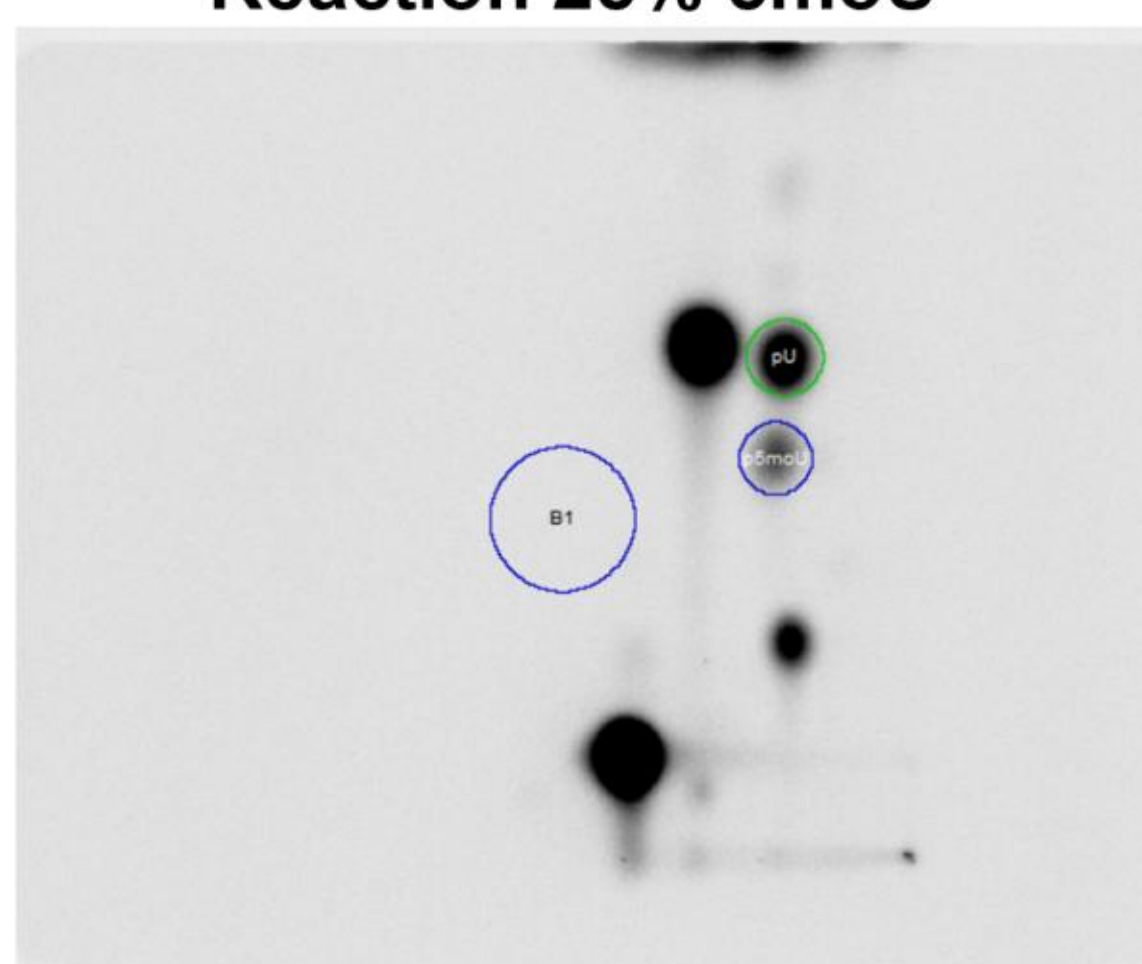

Index	Name	Volume INT*mm2	Adj. Vol. INT*mm2	% Adj. Vol.	Concentration	Density INT/mm2
1	p5moU	424192.1970	394715.2587	15.78 ←	N/A	93755.78325
2	pU	2139908.346	2106956.666	84.22	N/A	423092.5403
3	B1	117224.4790	0.000000000	N/A	N/A	6515.050163

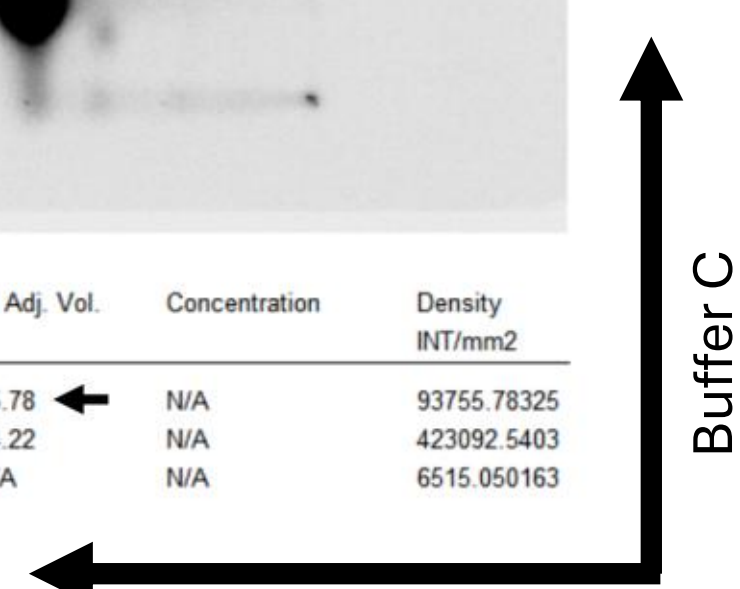

Figure S2. Uncropped 2D-TLC from Figure 2 and densitometry, Densitometry performed with QuantityOne (Biorad) following 2D-TLC (Figure 2) on separate reactions containing 50% or 25% 5moU during the IVT reaction.

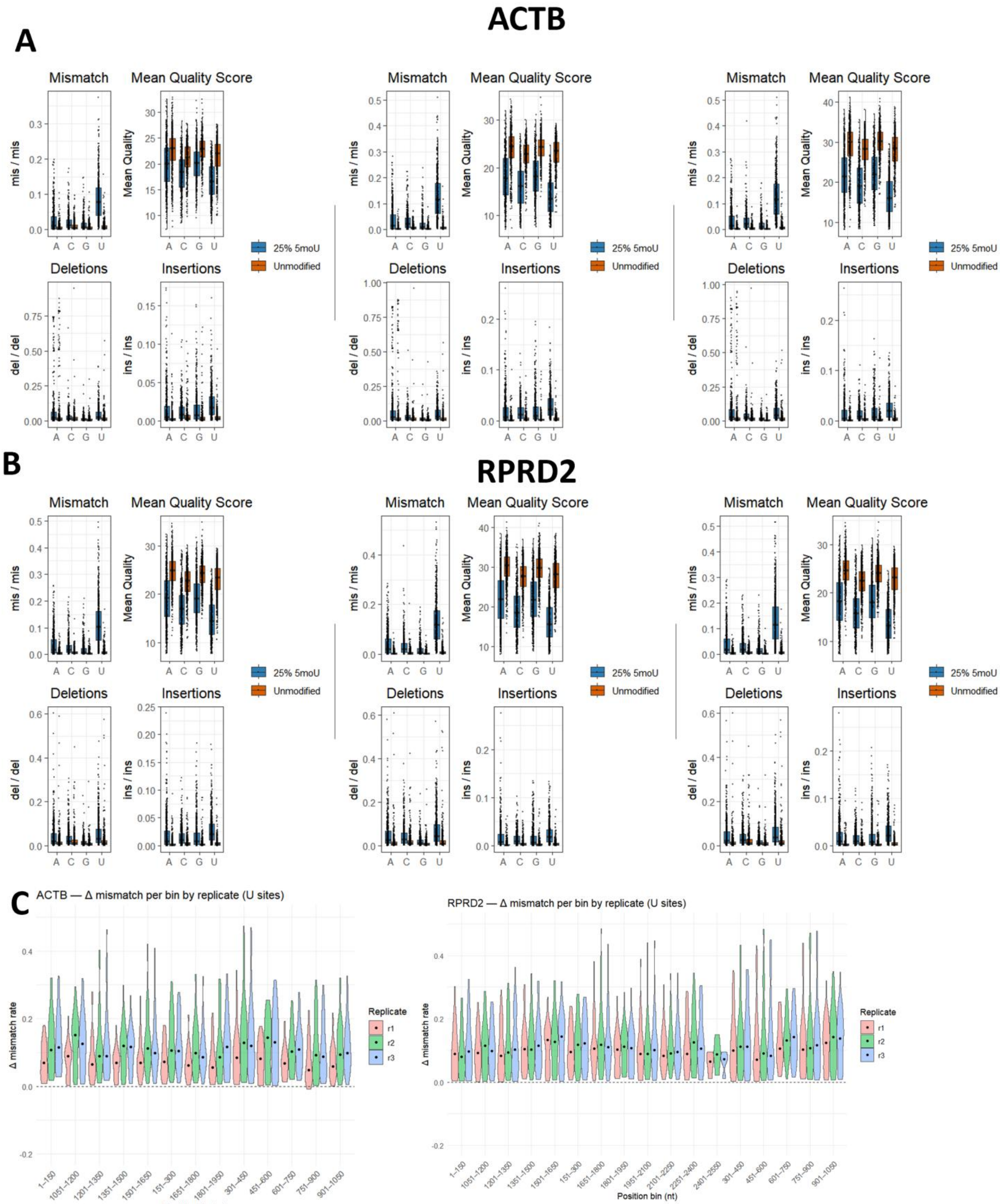
ACTB
A
Mismatch
Mean Quality Score
mis / mis
Mean Quality
25% 5moU
Unmodified
Deletions
Insertions
del / del
ins / ins
A C G U
B
RPRD2
C
ACTB — Δ mismatch per bin by replicate (U sites)
RPRD2 — Δ mismatch per bin by replicate (U sites)
Δ mismatch rate
Replicate
r1
r2
r3
Position bin (nt)

Figure S3. Individual Replicates of RNA004 sequenced unmodified and 25% 5moU-modified IVT. Box plots of rates of mismatch, deletion and insertion error rates, as well as mean quality scores, for each reference base from ACTB (A) and RPRD2 (B) mRNAs either unmodified or with 25% 5moU substitution. Values from each replicate sequenced are plotted separately. Violin plots (C) of Δ-mismatch rates at reference uracils in 150-ntd bins between 25% 5moU-modified and unmodified IVT for each replicate of ACTB or RPRD2 mRNA (N=3).

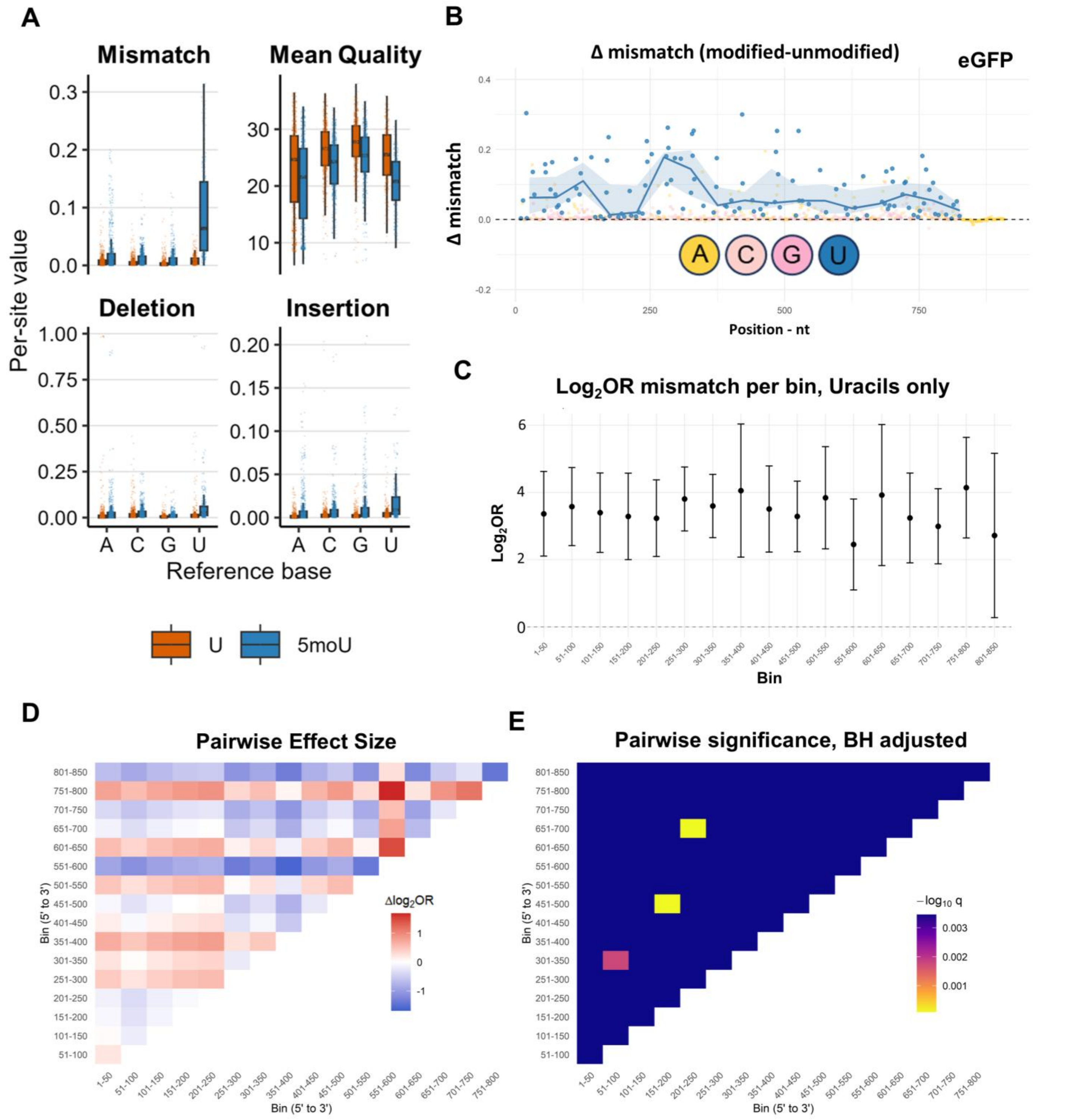

Figure S4. 25% 5moU-modified eGFP mRNA sequenced by DRS and compared to unmodified eGFP mRNA via our QC workflow. (A) Box plots of rates of mismatch, deletion and insertion error rates, as well as mean quality scores, for each reference base from eGFP mRNAs, averaged across three replicates. (B) Δ-mismatch rates at reference uracils in 50-ntd bins between 25% 5moU-modified and unmodified IVT (n=3). (C) Log_2 Odds Ratio for detected mismatch in each 50nt bin. (D&E) pairwise effect size (D) and BH-adjusted significance (E) of inter-bin differences in Log_2 odds ratio.

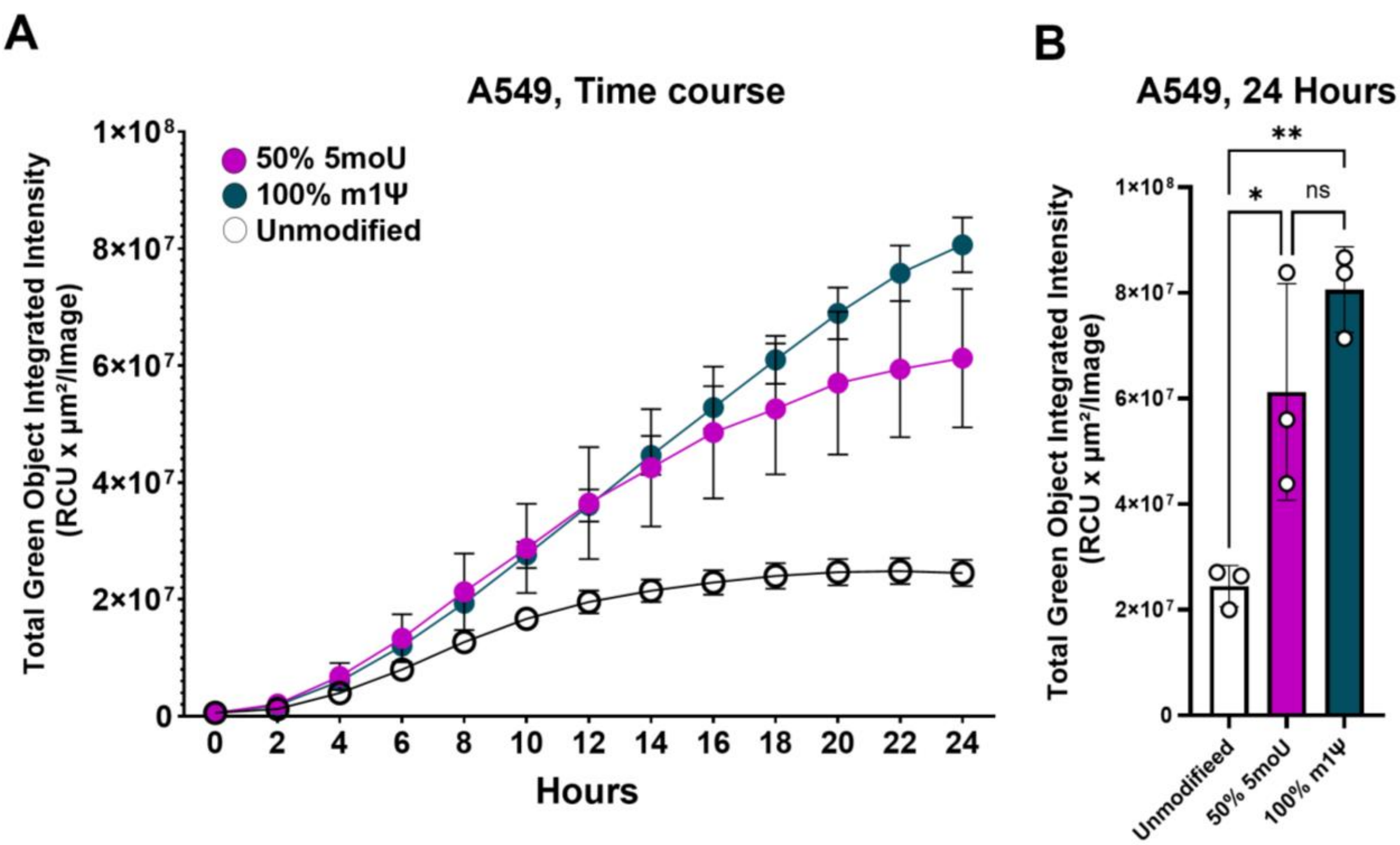

Figure S5. Comparison between U, $5moU_{50}$, and $m1\Psi_{100}$ eGFP expression in A549 cells. (A) Longitudinal expression data for eGFP fluorescence over 24hours after transfection with unmodified, 50% 5moU sub-stoichiometric, or m1Ψ 100% modified IVT mRNA. (B) The 24 hour timepoint, n=3, statistical test ANOVA with Tukey's HSD for pairwise comparisons. * = <0.05; ** = <0.005; ns = not significant.

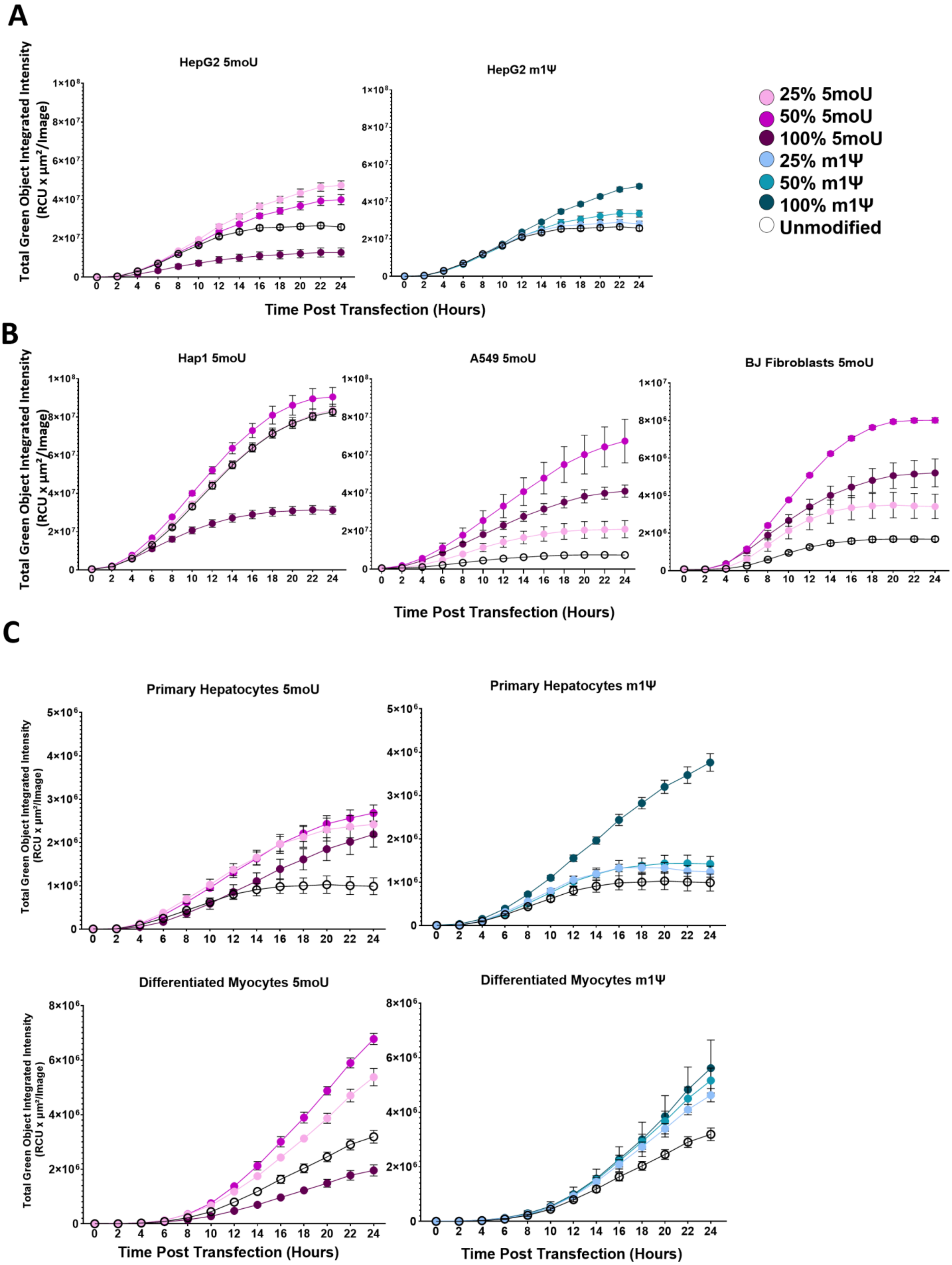

A
HepG2 5moU
HepG2 m1Ψ
Total Green Object Integrated Intensity (RCU x µm²/Image)
Time Post Transfection (Hours)
25% 5moU
50% 5moU
100% 5moU
25% m1Ψ
50% m1Ψ
100% m1Ψ
Unmodified
B
Hap1 5moU
A549 5moU
BJ Fibroblasts 5moU
C
Primary Hepatocytes 5moU
Primary Hepatocytes m1Ψ
Differentiated Myocytes 5moU
Differentiated Myocytes m1Ψ

Figure S6. Longitudinal expression data for eGFP fluorescence over 24hours after transfection with 5moU or m1Ψ-modified IVT mRNA, and at the stoichiometries defined in the legend. Fluorescence measurements were taken every two hours after transfection via live cell analysis (IncuCyte). (A) Hep G2 cells for 5moU and m1Ψ, (B) A548, Hap1, and BJ Fibroblast cells for 5moU, (C) Primary cell data for 5moU and m1Ψ. N=3

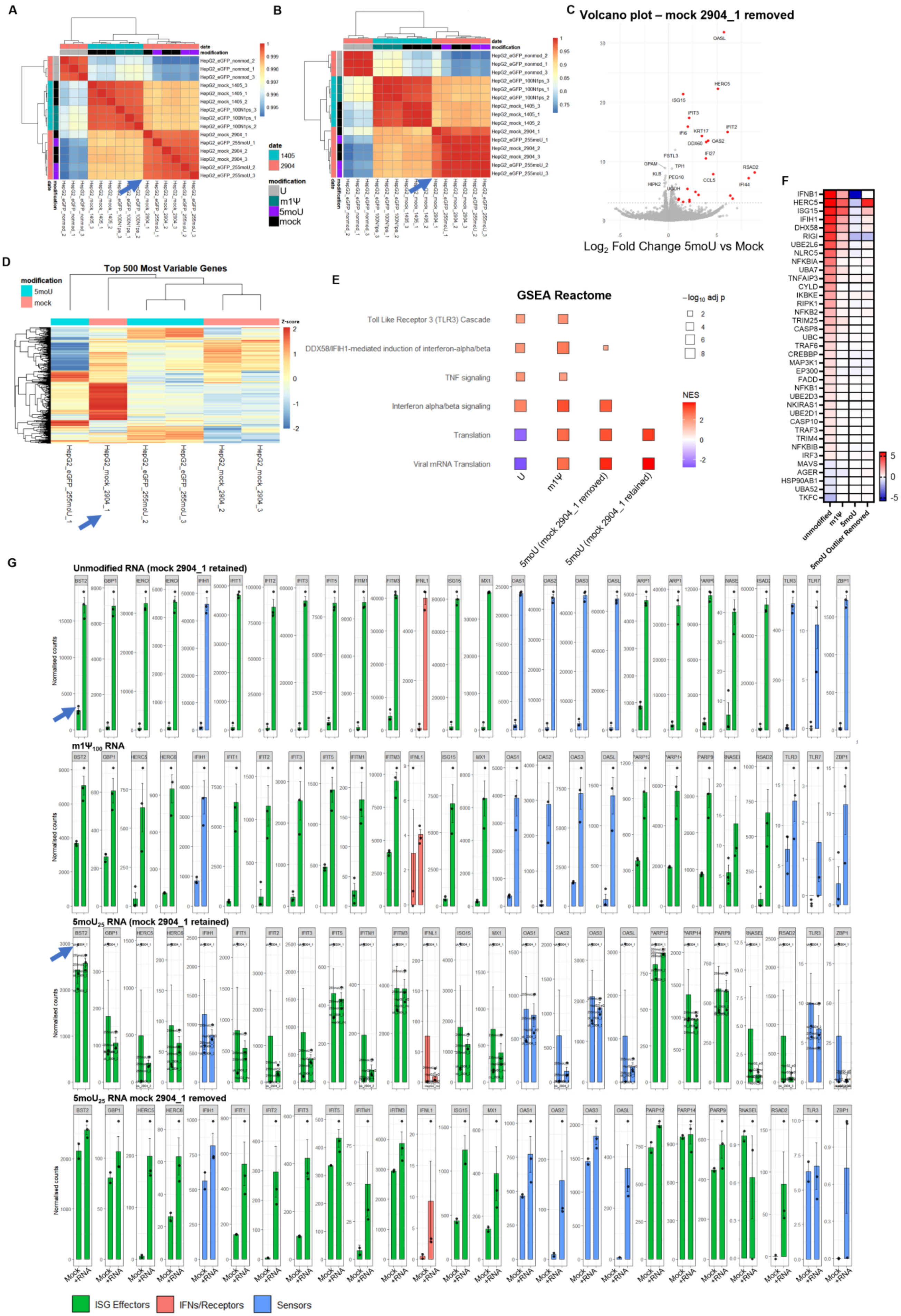
A
B
C
Volcano plot – mock 2904_1 removed
Log2 Fold Change 5moU vs Mock
D
Top 500 Most Variable Genes
modification
5moU
mock
E
GSEA Reactome
Toll Like Receptor 3 (TLR3) Cascade
DDX58/IFIH1-mediated induction of interferon-alpha/beta
TNF signaling
Interferon alpha/beta signaling
Translation
Viral mRNA Translation
U
m1Ψ
5moU (mock 2904_1 removed)
5moU (mock 2904_1 retained)
NES
F
unmodified
m1Ψ
5moU
5moU Outlier Removed
G
Unmodified RNA (mock 2904_1 retained)
m1Ψ100 RNA
5moU25 RNA (mock 2904_1 retained)
5moU25 RNA mock 2904_1 removed
Normalised counts
ISG Effectors
IFNs/Receptors
Sensors

Figure S7. Sample correlation and effects of sample removal.

(A) Pearson sample-sample correlation matrix computed using all genes after rlog transformation of DESeq2 results. (B) Correlation matrix computed using the 1000 most variable genes. Annotation colours indicate modification and date. (C) Volcano plot of differential expression with mock 2904_1 removed. (D) Hierarchical clustering of Z-score normalised, top 500 most variable genes when comparing only 5moU and Unmodified RNA, HepG2_mock_2904_1 noted with blue arrow. (E) Comparison of GSEA between all pairwise comparisons. (F) Heatmap showing log_2fold change across pairwise comparisons. (G) Plotting the normalised counts produced during DEseq2 analysis confirms the same mock sample is biasing $5moU_{25}$ comparisons (blue arrow) by reducing the effect size that is present following 5moU treatment. Removing this reveals that 5moU is not completely prevented from triggering the immune response.

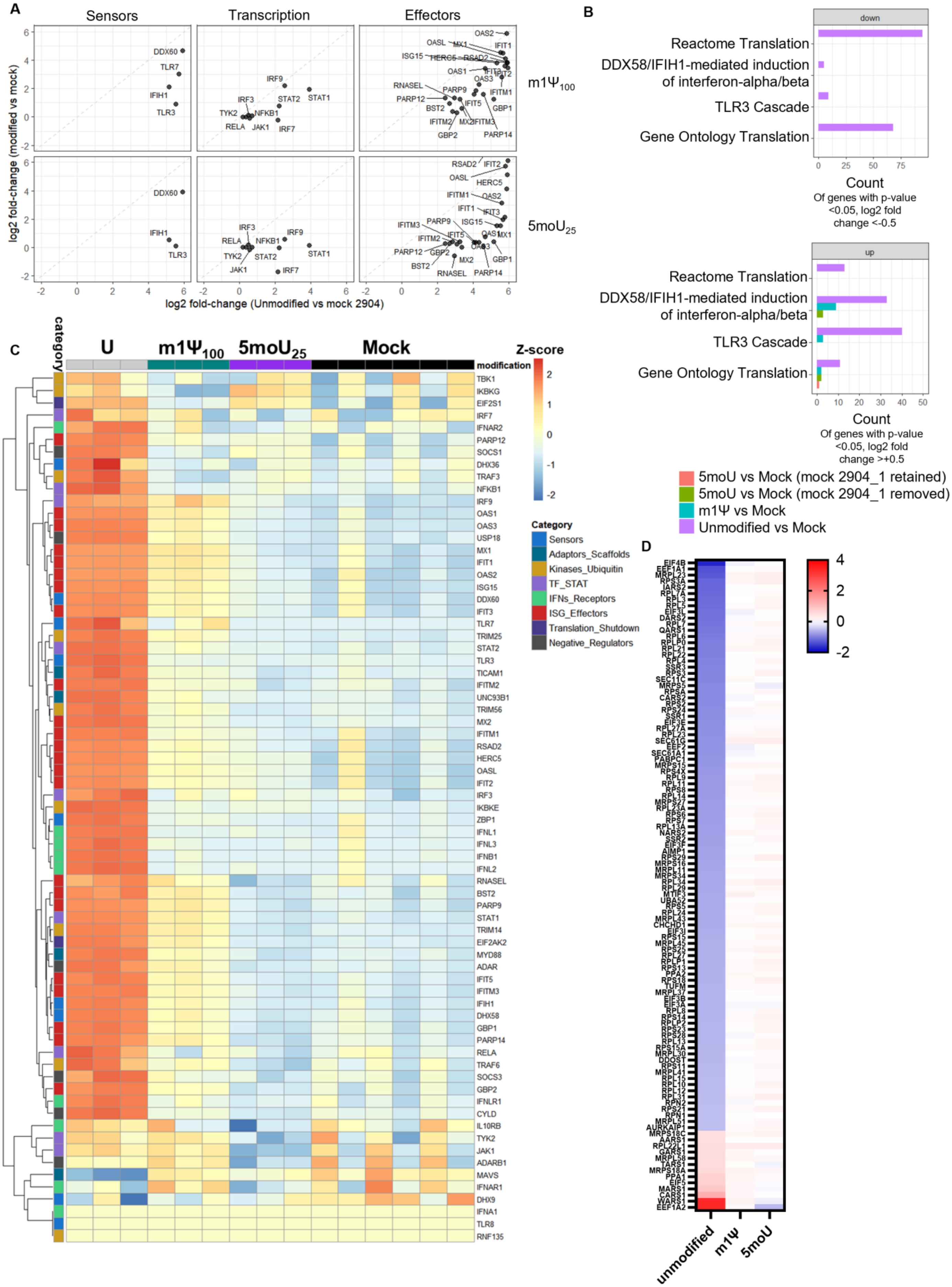

A
Sensors
Transcription
Effectors
m1Ψ100
5moU25
log2 fold-change (modified vs mock)
log2 fold-change (Unmodified vs mock 2904)
DDX60
TLR7
IFIH1
TLR3
IRF9
IRF3
STAT2
STAT1
TYK2
NFKB1
RELA
JAK1
IRF7
OAS2
OASL
MX1
IFIT1
ISG15
HERC5
RSAD2
OAS1
IFIT3
IFIT2
OAS3
RNASEL
PARP9
IFITM1
PARP12
IFIT5
GBP1
BST2
IFITM2
MX2
IFITM3
GBP2
PARP14
B
down
up
Reactome Translation
DDX58/IFIH1-mediated induction of interferon-alpha/beta
TLR3 Cascade
Gene Ontology Translation
Count
Of genes with p-value <0.05, log2 fold change <-0.5
Of genes with p-value <0.05, log2 fold change >+0.5
5moU vs Mock (mock 2904_1 retained)
5moU vs Mock (mock 2904_1 removed)
m1Ψ vs Mock
Unmodified vs Mock
C
category
U
m1Ψ100
5moU25
Mock
modification
Z-score
Category
Sensors
Adaptors_Scaffolds
Kinases_Ubiquitin
TF_STAT
IFNs_Receptors
ISG_Effectors
Translation_Shutdown
Negative_Regulators
TBK1
IKBKG
EIF2S1
IRF7
IFNAR2
PARP12
SOCS1
DHX36
TRAF3
NFKB1
IRF9
OAS1
OAS3
USP18
MX1
IFIT1
OAS2
ISG15
DDX60
IFIT3
TLR7
TRIM25
STAT2
TLR3
TICAM1
IFITM2
UNC93B1
TRIM56
MX2
IFITM1
RSAD2
HERC5
OASL
IFIT2
IRF3
IKBKE
ZBP1
IFNL1
IFNL3
IFNB1
IFNL2
RNASEL
BST2
PARP9
STAT1
TRIM14
EIF2AK2
MYD88
ADAR
IFIT5
IFITM3
IFIH1
DHX58
GBP1
PARP14
RELA
TRAF6
SOCS3
GBP2
IFNLR1
CYLD
IL10RB
TYK2
JAK1
ADARB1
MAVS
IFNAR1
DHX9
IFNA1
TLR8
RNF135
D
EIF4B
EEF1A2
unmodified
m1Ψ
5moU

Figure S8. (A) selected gene sets representing sensors, transcription, and effectors comparing modified mRNA with unmodified RNA differential gene expression analysis, where mock 2904_1 has been removed in $5moU_{25}$. (B) count of genes associated with specified terms in down- and up- regulated differentially expressed genes, with threshold of p-value <0.05 and log_2 fold change of +/- 0.5. (C) Full panel of foreign/innate RNA sensing genes for analysis, before sub-setting for Figure 4J-K and their expression, Z-score normalisation performed. (D) All genes included in Figure 4G heatmap, individually labelled.

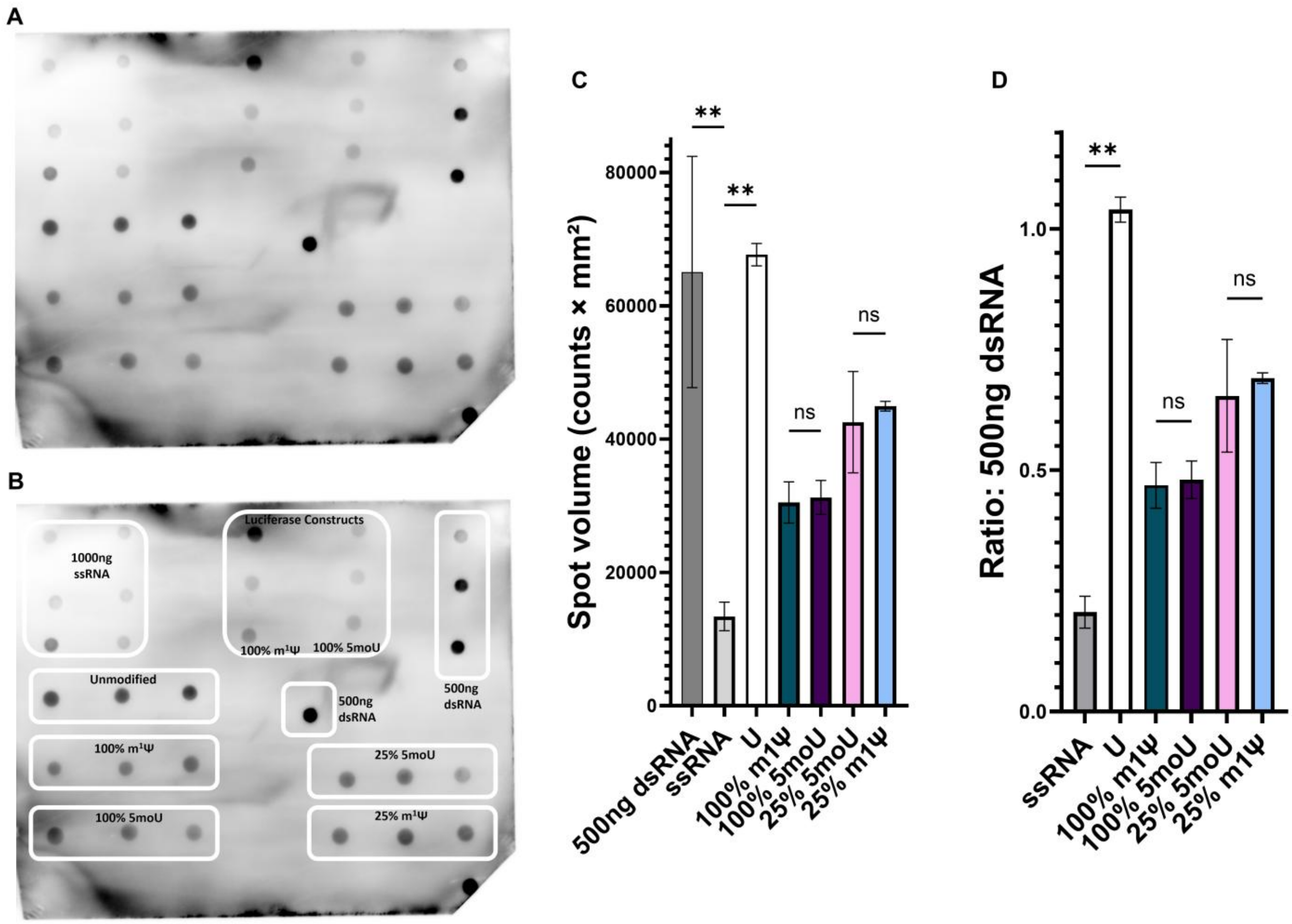

Figure S9. Dot blot assay measuring dsRNA content of in vitro transcripts and impact of modification. 500ng of dsRNA ladder, 1µg of ssRNA ladder, and 1µg of samples were analysed (A-D). (A) dot blot membrane chemiluminescent image (B) sample layout. (C&D) Quantification by spot volume (C) and a ratio of each to the 500ng dsRNA positive control (D). n=3, p-value calculated by Kruskal Wallis followed by Dunn's post-hoc, ** = 0.05.
